# Defensins of Grasses: A Systematic Review

**DOI:** 10.3390/biom10071029

**Published:** 2020-07-10

**Authors:** Tatyana I. Odintsova, Marina P. Slezina, Ekaterina A. Istomina

**Affiliations:** Laboratory of Molecular-Genetic Bases of Plant Immunity, Vavilov Institute of General Genetics RAS, 119333 Moscow, Russia; omey@list.ru (M.P.S.); mer06@yandex.ru (E.A.I.)

**Keywords:** antimicrobial peptides (AMPs), defensins, Poaceae, plant immunity, 3D structure modeling, in silico mining

## Abstract

The grass family (Poaceae) is one of the largest families of flowering plants, growing in all climatic zones of all continents, which includes species of exceptional economic importance. The high adaptability of grasses to adverse environmental factors implies the existence of efficient resistance mechanisms that involve the production of antimicrobial peptides (AMPs). Of plant AMPs, defensins represent one of the largest and best-studied families. Although wheat and barley seed γ-thionins were the first defensins isolated from plants, the functional characterization of grass defensins is still in its infancy. In this review, we summarize the current knowledge of the characterized defensins from cultivated and selected wild-growing grasses. For each species, isolation of defensins or production by heterologous expression, peptide structure, biological activity, and structure–function relationship are described, along with the gene expression data. We also provide our results on in silico mining of defensin-like sequences in the genomes of all described grass species and discuss their potential functions. The data presented will form the basis for elucidation of the mode of action of grass defensins and high adaptability of grasses to environmental stress and will provide novel potent molecules for practical use in medicine and agriculture.

## 1. Introduction

The grass family (Poaceae) is one of the largest and truly cosmopolitan families of flowering plants, including about 11,000 species that grow in all climatic zones of all continents [1,2,3]. Grasses are the first to occupy disturbed areas unsuitable for other plants. Some grasses are pernicious weeds that invade pastures and cropland. Grasses play a significant role in many plant communities and are the dominant vegetation of many habitats, such as grassland, steppes, and savannahs. The unique position of grasses among flowering plants is determined by their extremely high economic importance [2,4]. Cereals that produce edible grain, such as wheat, rye, maize, barley, rice, and oats, have been cultivated from ancient times and today play a crucial role in global food and feed production. Sugarcane grown in tropical countries is the source of sugar. Grasses are used as building materials in the manufacture of paper and biofuel. Some species are medicinal, while others are ornamental and grown to form lawns. Many grasses yield scented oils used in perfumery. Of no less importance are wild grasses, which form the basis of the vegetation of steppes, natural meadows, and pastures, and are used as fodder plants.

The widespread distribution of grasses suggests their high adaptation to adverse environmental factors of biotic and abiotic nature, which implies the existence of efficient resistance mechanisms. The most important stressful biotic factors that affect plants, causing their disease or even death, are pathogenic microorganisms, especially fungi [5]. While direct yield losses due to pathogens, weeds, and animals altogether range from 20 to 40% of global agricultural productivity, about 15% is lost due to diseases caused by pathogenic microorganisms [6,7]. The study of the molecular components and mechanisms of the innate immune system of grasses is necessary to understand their effective adaptation to environmental conditions and to develop new strategies to increase the disease resistance of species of exceptional economic significance.

## 2. Antimicrobial Peptides

Plants have evolved a complex, multilevel immune system to combat an immense variety of pathogens, which differ in terms of nutrition type and plant colonization strategy [8]. This system includes physical barriers to the penetration and spread of pathogenic microorganisms and a whole arsenal of defense compounds that inhibit their growth and development.

Among plant defense molecules, antimicrobial peptides (AMPs) play a pivotal role [9,10,11,12,13,14,15,16,17,18,19,20]. These gene-encoded defense molecules display high structural diversity and broad-spectrum antimicrobial activity. Several families have been identified, including defensins, thionins, lipid-transfer proteins, snakins, hevein-like peptides, knottins, and hairpinins. A unique and important feature of AMPs is their ability to inhibit the growth and development of a wide range of pathogens rather than a narrow range of highly specialized races. Another major distinguishing feature of AMPs is that they are not prone to resistance breakdown, since their mode of action exploits general but essential structural components of pathogens, such as the cell membrane, and in many cases they have additional targets within cells. Thus, the use of AMPs to increase plant resistance through genetic transformation is extremely promising [21].

Despite considerable success having been achieved under laboratory conditions in genetic transformation of plants using plant AMP genes as transgenes, the potential of plant AMPs is not fully exploited. AMPs of grasses remain poorly studied. Single AMPs have been isolated and functionally characterized from cultivated cereals. Wild grasses that are perfectly adapted to diverse environmental conditions are even less studied, although they represent a valuable source of AMPs and other defense molecules. Unlike cultivated species, wild plants are more resistant to pathogens and abiotic stress. As shown by our research on wild-growing plants and weeds belonging to different families (*Stellaria media* L., *Echinochloa crus-galli* (L.) Beauv., *Leymus arenarius* (L.) Hochst., *Taraxacum officinale* L.), their increased resistance to pathogens is largely associated with the presence of highly active AMPs [22,23,24,25,26,27,28,29,30].

## 3. Plant Defensins: General Characteristics

Defensins belong to one of the largest and most ancient AMP families. They are widely distributed throughout the plant and animal kingdoms and found in invertebrates, birds, mammals and plants, and even in fungi [31,32,33,34,35,36]. In the completely sequenced plant genomes, dozens of defensin-like sequences were discovered [37,38]. However, the functions of the vast majority of them are poorly understood. Plant defensins are small (45‒54 amino acids) cationic peptides with a characteristic 8-cysteine motif. All cysteine residues are engaged in 4 disulphide bonds [31]. Petunia defensins, which possess 5 disulphide bonds, are an exception [32]. Despite considerable sequence variation in addition to conserved cysteine residues, the three-dimensional structure of defensins is similar and involves an α-helix and a triple-stranded antiparallel β-sheet. The main structural feature of the defensin molecule is the so-called cysteine-stabilized α-helix β-sheet motif (CSαβ), in which two cysteine residues separated by one turn of the α-helix are connected to two cysteines, which are located a single amino acid apart in the third β-strand [31]. Recently, plant defensins have been assigned to the cis-defensin superfamily, in which two parallel disulphide bonds connect the third β-strand to the α-helix [39]. Defensins are synthesized as precursor proteins [32]. The precursors of class 1 defensins consist of a signal peptide and a mature defensin domain, while the precursors of class 2 defensins have an additional C-terminal prodomain, which is involved in targeting the vacuoles and detoxification of the peptides in plant cells [40]. Many plant defensins have been isolated from seeds, however they are also found in other plant organs, such as leaves, flowers, and fruits. Some members of the family are induced upon pathogen attack or other environmental stimuli, while others are constitutively expressed in specific tissues or organs [32].

In vitro studies have revealed a number of activities of plant defensins [41]. Most plant defensins exhibit antifungal activity in vitro [42,43]. According to their effect on fungi, they are separated into two groups: the morphogenetic defensins cause morphological changes in fungi, such as hyperbranching, while the nonmorphogenetic defensins only induce growth inhibition [31]. Some defensins inhibit growth of bacteria [44]. The antimicrobial activity of defensins detected by in vitro studies suggests their role in protection of plants against pathogenic microorganisms, a role supported by enhanced pathogen resistance of transgenic plants expressing defensin genes [32,45]. In addition to antimicrobial activity, the inhibition of protein synthesis in vitro [46,47]; inhibition of enzymes (proteases and α-amylases) [48,49,50,51]; blockage of L-type Ca^2+^ [52], sodium, and potassium channels [53,54]; cytotoxic activity towards human tumor cells and plant cells have also been reported for plant defensins [55,56,57,58]. The participation of defensins in resistance to abiotic stress and developmental processes was shown for some members of the family [32,35,39,45,59,60].

Although wheat and barley seed γ-thionins were the first defensins isolated from plants, the functional characterization of grass defensins is still in its infancy. In this review, we summarize the current knowledge of the defensins from cultivated and selected wild-growing grasses. For each plant species, isolation of defensins or production by heterologous expression, peptide structure, biological activity, and structure–function relationships (where available) are described, and gene expression data are provided. It is worth noting that isolation of defensins from plants by protein chemistry methods is a laborious and time-consuming task, which is currently being replaced by gene cloning and recombinant production. However, the usefulness of direct isolation of peptides from plant tissues cannot be underestimated, because only this approach provides reliable information on the mature peptide sequence (avoiding predictions of the signal peptide and the C-terminal prodomain (if present) cleavage sites) and on the peptide’s expression. We also provide our data on in silico mining of the defensin-like (DEFL) sequences with a cysteine motif of “classical” defensins CX(4,25)CX(2,12)CX(3,4)CX(3,17)CX(4,32)CXCX(1,6)C as defined by Silverstein et al. [38] in the genomes and transcriptomes of all of the described cultivated and some wild-growing grass species. Finally, we summarize the bioinformatics-based data on the molecular diversity of grass defensins and discuss their functions.

## 4. Defensins of Grasses

### 4.1. Wheat

Defensins were isolated and sequenced by protein chemistry methods from kernels of three wheat species that differ in ploidy levels—a tetraploid wheat *Triticum turgidum* L. (genome composition AABB) cv. Senatore Capelli [61], a hexaploid wheat species *T. kiharae* Dorof. et Migush. (genome composition AAGGDD), and a diploid species *T. monococcum* L. (genome composition AA) [62]. Furthermore, five defensins from *T. kiharae*, one defensin from *T. aestivum* L., and several defensins from *T. urartu* Thumanjan ex Gandilyan, *T. boeoticum* Boiss. and *Aegilops tauschii* Coss. were N-terminally sequenced [63,64,65]. The structure of the TAD1 defensin from *T. aestivum* was deduced from the cDNA clone [66].

#### 4.1.1. γ-Purothionins of *T. turgidum*

##### Isolation

Wheat defensins (initially named γ-purothionins) were the first plant defensins isolated by Collila et al. [61] from the grain of the durum wheat, *T. turgidum*. The isolation procedure included several extraction steps followed by two reversed-phase high-perfomance liquid chromatography (RP-HPLC) separations. First, the chloroform–methanol fraction of the wheat endosperm was obtained, followed by extraction with 0.5 M NaCl, precipitation with trichloroacetic acid to 15% final concentration, and extraction with 50 mM ammonium bicarbonate. The bicarbonate-insoluble fraction was separated by RP-HPLC on a Nucleosil C4 silica column in acetonitrile gradient (10–35%) containing 0.1% trifluoroacetic acid (TFA). Then, γ-thionins were purified by rechromatography on the same column using a shallower acetonitrile gradient. As a result, two peptides named γ_1_-P and γ_2_-P were isolated.

##### Amino Acid Sequencing and Primary Structure Analysis

**Figure 1:**
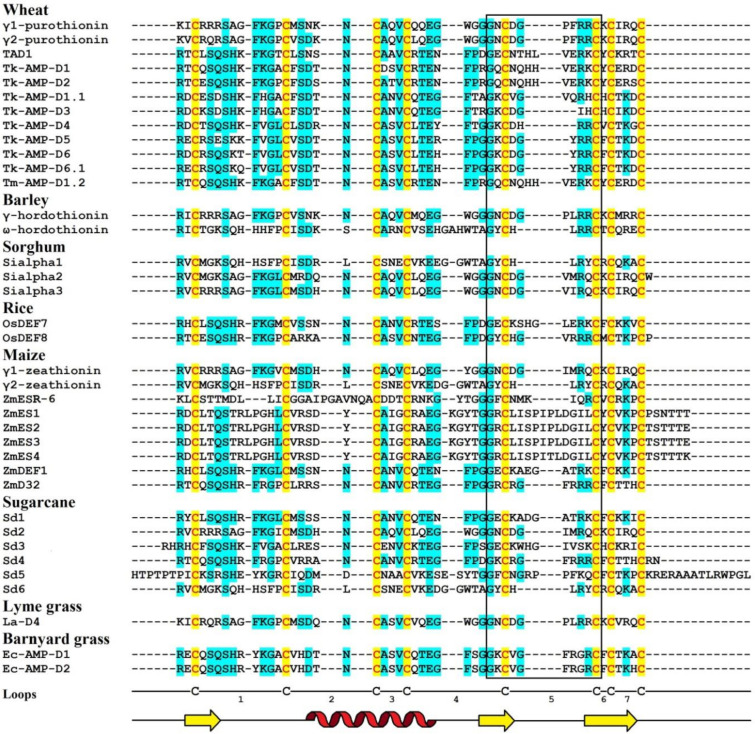
Amino acid sequences of characterized grass defensins. Cysteine residues are shaded yellow, while identical amino acids are shaded cyan. Loops defined as regions between cysteine residues [41] and the position of β-strands (yellow) and the α-helix (red) are indicated below the figure. A black frame borders the γ-core.

Amino acid sequences of γ_1_-P and γ_2_-P were determined by automated Edman degradation of the intact molecules and of the chymotryptic peptides (Figure 1) [61]. The peptides are very similar in their amino acid sequences, differing by only 5 amino acid residues, including 2 conserved substitutions. Variant amino acid residues are located in the N-terminal half of the molecule, in loops 1, 2, and 4 (loops are regions between the adjacent cysteine residues as defined by van der Weerden and Anderson [41]). Both peptides consist of 47 amino acid residues, 8 of which are cysteines occupying the same positions in both peptides, thus forming the cysteine motif as follows: CX10CX5CX3CX9CX6CXCX3C. The calculated molecular masses of γ_1_-P and γ_2_-P are 5239 and 5151 Da, respectively. The main characteristics of the peptides are summarized in Table 1. Comparison of the γ_1_-P and γ_2_-P amino acid sequences with classical α- and β-thionins disclosed rather low sequence similarity (32% identical residues) and a different cysteine motif (the cysteine motif in α- and β-thionins is CCX7CX3CX8CX3CXCX7C) [61]. The dissimilarity of γ-purothionins with thionins was further confirmed by Nuclear Magnetic Resonance (NMR) spectroscopy.

##### 3D Structure Analysis

**Figure 2:**
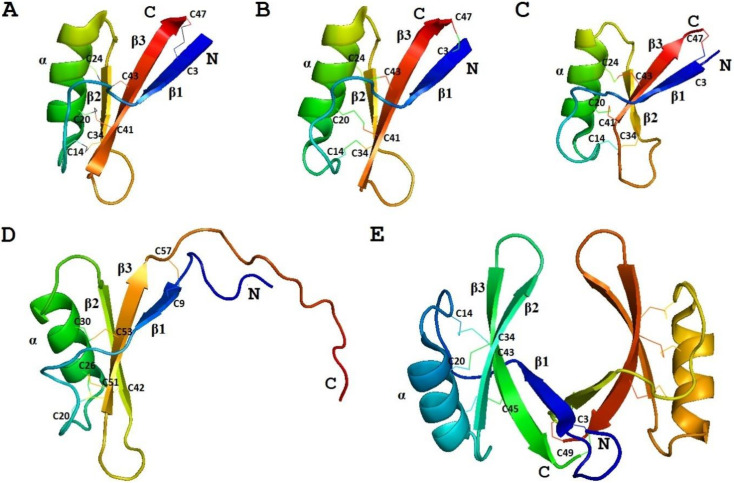
The 3D structure of defensins: (**A**) wheat γ_1_-purothionin (PDB 1GPS), (**B**) barley γ-hordothionin (PDB 1GPT), (**C**) maize ZmD32 (PDB 6DMZ), (**D**) sugarcane Sd5 (PDB 2KSK), (**E**) rice OsAFP1 (PDB 6LCQ). Disulfide bonds are shown by thin lines. The N-and C-termini are indicated by N and C, respectively. Secondary structure elements are denoted by α for α-helix and β for β-strand.

The 3D structure of γ_1_-P was solved by NMR (Figure 2) [77]. The fold of the peptide is characterized by a triple-stranded antiparallel β-sheet, an α-helix, and connecting loops. The compact structure is held together by 4 disulphide bridges. Positively charged residues are unevenly distributed in the molecule. The 3D structure of the γ-purothionin is quite different from that of classical thionins resembling the Greek letter Γ, in which the long arm is formed by two antiparallel α-helices, while the short one is formed by two parallel β-strands [78]. Based on these dissimilarities between α- and β-thionins, and γ-purothionins and the similarity of γ-purothionins to insect defensins, they were renamed “plant defensins” [79].

##### Biological Activity

The γ_1_-P was assayed against five fungi (*Botrytis cinerea*, *Cladosporium sphaerospermum*, *Fusarium culmorum*, *Penicillium digitatum*, *Trichoderma viride*) and found inactive at concentrations up to 200 µg/mL [80]. The γ_2_-P homologue named AFP-1, which was isolated from wheat primed seeds, was assayed against another panel of pathogens: *F. graminearum*, *Phytophthora infestans*, *Stagonospora nodorum*, *Magnaporthe grisea* and *Verticillium dahliae* [63]. AFP-1 was active against four of them, except for *M. grisea,* however at high concentrations. The half maximal inhibitory concentration (IC_50_) values for *F. graminearum*, *P. infestans*, *S. nodorum*, and *V. dahliae* were 270, 70, 140, and 70 µg/mL, respectively.

#### 4.1.2. Defensins of *T. kiharae* and *T. monococcum*

##### Isolation

Eight defensins named Tk-AMP-D1, Tk-AMP-D1.1, Tk-AMP-D2, Tk-AMP-D3, Tk-AMP-D4, Tk-AMP-D5, Tk-AMP-D6, and Tk-AMP-D6.1, and one defensin Tm-AMP-D1.2 were isolated from *T. kiharae* and *T. monococcum* seeds [62], respectively, by a combination of acidic extraction and three HPLC separations [64,81]. Flour was extracted with a mixture of 1% (*v*/*v*) TFA, 1 M HCl, 5% formic acid, and 1% (*w*/*v*) NaCl in the presence of pepstatin A. The protein–peptide fraction was further separated by chromatography on a HiTrap Heparin HP column (GE Healthcare, UK). The D-defensin-containing fraction was eluted with 100-mM NaCl in 10 mM tris-HCl buffer (pH 7.5), while Tk-AMP-γ and Tk-AMP-ω were recovered from the column with 500 mM NaCl. Both fractions were further separated by size-exclusion chromatography on a Superdex Peptide HR 10/30 column (GE Healthcare, UK) followed by RP-HPLC on a Vydac C18 column with a linear acetonitrile gradient.

##### Amino Acid Sequencing and Primary Structure Analysis

The complete amino acid sequences of Tk-AMP-D and Tm-AMP-D1.2 defensins were determined by automated Edman degradation of reduced and alkylated peptides (Figure 1) [62]. For D1 and D6, additional cleavage with Glu-C endoproteinase and sequencing of the peptides produced were carried out. A total of nine defensins were isolated from *T. kiharae* and *T. monococcum* seeds. Their main characteristics are presented in Table 1. The peptides differ in size—from 49 amino acid residues in D1, D2, and D1.2, to 45 amino acid residues in D3 and D4. The variation in size is due to different lengths of loop 5. The charge of D defensins at pH 7.0 is also variable—from 0 in D3 to +3 in D2, D4, D5, and D1.2. One defensin, D1.1, is negatively charged. The charge of D defensins is significantly lower than that of γ-purothionins (Table 1). Sequence analysis revealed high similarity among D defensins, demonstrating that they belong to a family of closely related peptides (Figure 1).

##### Biological Activity

The biological activity of Tk-AMP-D1 and Tk-AMP-D6 was tested against four pathogenic fungi, namely *Fusarium graminearum*, *Colletotrichum graminicola*, *F. verticillioides*, and *Diplodia maydis*, using a 4-point scale, as suggested by Duvick et al. [82]. The peptide concentrations were ≤30 µg/mL [83]. The antifungal activity of Tk-AMP-D1 against *F. graminearum* was 1.5. Both peptides displayed rather weak antifungal activity against *F. verticillioides* and scored 1 and 0.5 for Tk-AMP-D1 and Tk-AMP-D6, respectively, at the highest tested concentration (30 µg/mL). Both peptides were inactive against *C. graminicola* and *D. maydis* [83].

#### 4.1.3. DEFLs of *T. kiharae* Seedlings Analyzed by RNA-Seq

*T. kiharae* defensins were further studied by transcriptome analysis of wheat seedlings using high-throughput RNA-seq technology [84]. Using a combination of hidden Markov models and regular expressions, 52 sequences encoding defensin-like precursors named group 1 DEFLs were identified. All of them consisted of a signal peptide and a mature peptide region with the 8-cysteine motif characteristic of classical defensins [38]. In some precursors, the mature peptide regions were identical, leaving 35 unique defensins (Figure S1). The length of defensins varied from 46 to 60 residues and their predicted isoelectric point (pI) values ranged from 7.23 to 9.93 [84].

##### Expression Analysis of DEFL Genes

Expression of DEFL genes was studied upon infection with *F. oxysporum*, treatment with the *F. sambucinum* FS-94 elicitors triggering induced resistance, and upon challenge inoculation with *F. oxysporum* of elicitor-pretreated seedlings [84]. Transcriptome analysis showed that all treatments changed the expression of different sets of group 1 DEFLs [84]. Infection with *F. oxysporum* up-regulated approximately two-fold expression of four DEFLs (DEFLs 1-11, -12, -32, -43), providing indirect evidence for their involvement in protection against this fungus (Figure S1). Of these, the antifungal activity was shown for DEFL1-43 (see below). From sequence similarity between DEFLs 1-11 and 1-32 and the barley ω-hordothionin, an inhibitor of protein synthesis in pro- and eukaryotic cell-free systems (see below), we can suggest similar functions for *T. kiharae* peptides. DEFL1-12 shares similarity with γ_1_- and γ_2_-purothionins, the latter of which is weakly antifungal (see above). Treatment with the elicitors up-regulated another set of genes (DEFLs 1-1, -14, -16, -29, -30, -34, -36, -39, -50) [84]. Their expression level increased from 2.5- to 19-fold. These genes are likely to be involved in the activation of the elicitor-triggered defense response. Of elicitor-activated genes, DEFL1-16 is highly similar to the maize defensin ZmD32, with the potent antifungal activity (see below) suggesting the same role for the wheat peptide [72]. In plants displaying induced resistance, the following set of DEFL genes were up-regulated (DEFLs 1-1, -2, -11, -12, -16, -25, -28, -32, -34, -36, -39, -41, -43 -50), including all of those induced by *F. oxysporum* infection and several genes up-regulated by the elicitors. Four remaining DEFL genes were primed by the elicitors (DEFLs 1-2, -25, -28, and -41). Up-regulated group 1 DEFLs, together with other defense proteins and peptides, may contribute to the enhanced resistance state exhibited by the elicitor-pretreated wheat seedlings.

##### Biological Activity

Only DEFL1-43 up-regulated by *F. oxysporum* infection and challenge inoculation of elicitor-pretreated wheat seedlings (named Tk-AMP-γ3 in seeds [64]) was assayed in vitro for antifungal activity against *F. graminearum*, *F. verticillioides*, *C. graminicola*, and *D. maydis* [83]. Its inhibitory activities against *F. graminearum* and *F. verticillioides* at the concentration of 30 µg/mL were rated 1 and 2, respectively, using the 4-point scale of Duvick et al. [82]. The peptide was inactive against *C. graminicola* and *D. maydis* at concentrations ≤30 µg/mL. Thus, the antifungal activity of DEFL-43 was similar to that of seed defensins Tk-AMP-D1 and Tk-AMP-D6, which is not surprising considering the high sequence similarity between these peptides (Figure S1).

#### 4.1.4. Defensin TAD1 of *T. aestivum*

The primary structure of the defensin TAD1 precursor protein was deduced from the nucleotide sequence of the cDNA clone induced after cold acclimation in winter wheat [66].

##### Sequence Analysis

The precursor consists of 82 amino acid residues, with a signal peptide of 33 residues and the mature peptide domain of 49 amino acid residues [66]. The amino acid sequence and main characteristics of TAD1 are presented in Figure 1 and Table 1, respectively. The TAD1 amino acid sequence is nearly identical to that of DEFL1-17. The variation concerns a single amino acid residue, which is Ala22 in TAD1 and Gly22 in DEFL1-17.

##### Recombinant Production

TAD1 was produced in *Escherichia coli* cells as a recombinant fusion protein with glutathione S-transferase (GST), purified by affinity chromatography on a glutathione–Sepharose 4B column and on-column-digested with PreScission protease. The eluted recombinant peptide TAD1 was purified by filtration using Centricon YM-30 spincolumn [66].

##### Biological Activity

Koike et al. [66] assayed the antimicrobial activity of the recombinant peptide TAD1 by in vitro tests against *Pseudomonas cichorii* SPC900, a pathogenic Gram-negative bacterium with a broad host range, which is spread in warm and humid areas, and showed the inhibitory effect of the peptide on the bacterial growth at IC_50_ = 25 µg/mL.

Studies of the antifungal activity of the recombinant TAD1 against the snow mold fungus *Typhula ishikariensis* in liquid culture at the peptide concentrations of ≤100 µg/mL revealed growth inhibition in a dose-dependent manner [85]. At a concentration of 10 µg/mL, the morphology of hyphae was affected and balloon-like cells appeared at the tips of the hyphae. At higher peptide concentrations (50‒100 µg/mL), the burst of hyphae was recorded. Further studies of resistance of the transgenic wheat plants overexpressing *TAD1* by inoculation of detached leaves and measurement of leaf lesion areas showed enhanced resistance to *T. ishikariensis* and to *F. graminearum* causing head blight [85].

No antifreeze activity was detected by observation of ice crystal morphology in the presence of the recombinant TAD1 peptide [66].

##### Expression Analysis

Abiotic stress, such as low temperatures, induced expression of *TAD1*. TAD1 mRNA was shown to accumulate at high levels after one day of cold acclimation in the crown tissue of wheat; this high expression was preserved during two weeks of cold acclimation. The gene was also induced in young seedlings exposed to low temperatures [66]. Exogenous abscisic acid, salicylic acid, and methyl jasmonate did not induce *TAD1* expression [66]. The authors suggest that *TAD1* participates in cold-induced resistance to pathogens involving defense signaling pathways independent of salicylic acid and methyl jasmonate [66,85].

#### 4.1.5. Defensin-Like Peptides Identified in *T. aestivum* and Related Species by In Silico Mining

In order to identify defensin-like sequences in the wheat *T. aestivum* and related species (*T. turgidum*, *T. urartu* and *A. tauschii*), we carried out analysis of the NCBI (https://www.ncbi.nlm.nih.gov/) and UniProt (https://www.uniprot.org/) databases and retrieved sequences annotated as putative defensins and defensin-like proteins. The genus *Triticum* comprises species of different ploidy levels from diploid (2n = 14) to hexaploid (2n = 42). Tetraploid (2n = 28) and hexaploid wheat species are natural allopolyploids, which arose from interspecific hybridization between species of the *Triticum* and *Aegilops* genera [86,87]. It has been generally recognized that the polyploid wheat species form two evolutionary lineages—*T. turgidum* (AABB) and *T. aestivum* (AABBDD) constitute one lineage, while *T. timopheevii* Zhuk. (AAGG) and *T. zhukovskyi* Menabde & Erizin form the other. Wild tetraploid wheat *T. turgidum* ssp. *dicoccoides* (genome composition AABB) originated by hybridization of a wild diploid wheat with the genome formula AA with the B genome donor. The hexaploid *T. aestivum* (genome composition AABBDD) originated by hybridization of *T. turgidum* with the D genome donor. The hexaploid wheat *T. kiharae* is a synthetic allopolyploid, which arose from hybridization between *T. timopheevii* and *A. tauschii. A. tauschii* was unanimously recognized as the D genome donor to polyploid wheat genomes. Most data indicate that *A. speltoides* Tausch. contributed the B genome to bread and durum wheats. *T. urartu* is accepted as the A genome donor in both lineages.

**Figure 3:**
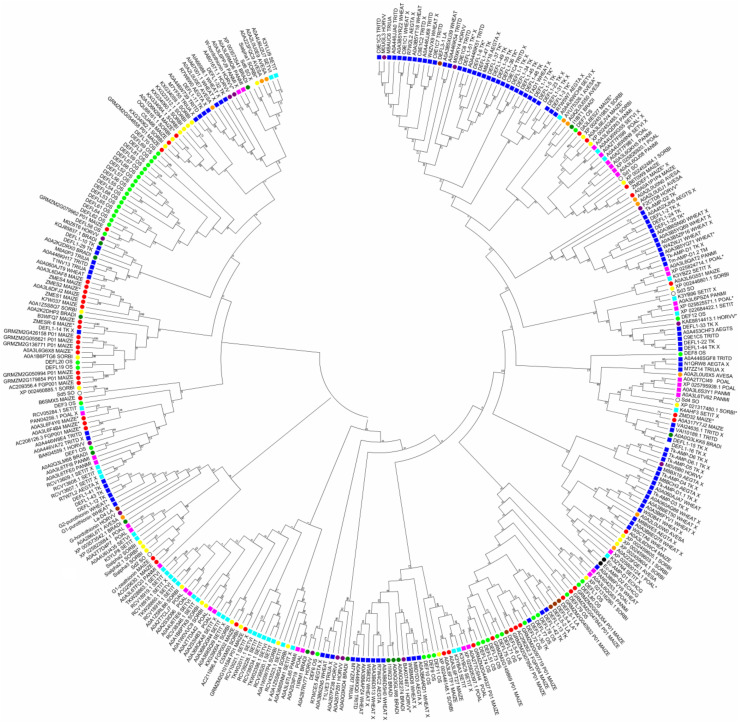
A phylogenetic tree of the grass defensins and defensin-like (DEFL) peptides. The amino acid sequences of the mature DEFLs were used for the phylogenetic tree construction with the MEGA X software [88]. DEFLs of *Triticum* spp. and *Aegilops tauschii* are marked with blue squares, while those of *Panicum* spp. and *Setaria* spp. are marked with pink and cyan squares, respectively. DEFLs of *O. sativa* are marked with light green circles, *Z. mays* with red, *Sorghum bicolor* with yellow, *H. vulgare* with violet, *Avena sativa* with orange, *B. distachyon* with green, *L. arenarius* with brown, *E. crus-galli* with black, and *Saccharum* spp. with white circles. The following abbreviations are used: TK—*T. kiharae*; TRITD—*T. turgidum*; TRIUA—*T. urartu*; WHEAT—*T. aestivum*; AEGTS—*Aegilops tauschii* ssp. *strangulata*; AEGTA—*A. tauschii* ssp. *tauschii*; AVESA—*Avena sativa*; SETIT—*Setaria italica*; SETVI—*S. viridis*; POAL—*Panicum hallii*; PANMI—*P. miliaceum*; MAIZE—*Z. mays*; LA—*L. arenarius*; OS—*O. sativa*; HORVV—*H. vulgare*; SORBI—*Sorghum biolor*; BRADI—*B. distachyon*; SO—*Saccharum* spp. Note: * and X mark DEFL sequences with identical mature peptides within (*) and between (X) grass species (see Tables S3 and S4). Bootstrapping was carried out 1000 times to obtain support values for each branch.

By database analysis, we discovered 69 DEFL precursor sequences in *T. aestivum*, 37 DEFLs in *T. turgidum*, 16 DEFLs in *T. urartu*, and 44 DEFLs in *A. tauschii* (23 in *A. tauschii* spp. *strangulata* and 21 in *A. tauschii* spp. *tauschii*) (Tables S1 and S2, Figures S2–S6). The number of DEFL sequences is in agreement with the ploidy level of the species. Similarly to *T. kiharae*, precursors with identical mature peptide domains arising from duplications occurring in the course of defensin gene evolution were discovered in each species, except for *T. urartu* (Table S3). Sequence comparison disclosed high sequence similarity and even identity between DEFL proteins of polyploid wheat species with those of subgenome donors, supporting their origin from *A. tauschii* or *T. urartu* (Table S4). Note that more sequences of polyploid species showed similarity to *Aegilops* defensins than to those of *T. urartu*, which reflects the more recent acquisition of the D genome by a tetraploid progenitor of hexaploids. A phylogenetic tree based on grass defensin mature peptide sequences constructed using the neighbor-joining method shows that defensins of *Triticum* and *Aegilops* species occur in the same clades, and occasionally together with closely related *Hordeum vulgare* L., *L. arenarius*, and *Brachypodium distachyon* (L.) P.Beauv. (Figure 3).

### 4.2. Barley

Two defensins named γ-hordothionin (γ-H) and ω-hordothionin (ω-H), which were also initially assigned to a novel subclass of thionins together with γ-purothionins, were isolated from barley seeds by Mendez et al. [46,47].

#### 4.2.1. Hordothionins

##### Isolation

The salt-soluble fraction was obtained from barley endosperm and further extracted with 50 mM ammonium bicarbonate. The bicarbonate-insoluble fraction was separated by RP-HPLC on a semi-preparative Nucleosil C4 silica column in acetonitrile gradient (10‒20%) containing 0.1% TFA [46].

##### Amino Acid Sequencing and Primary Structure Analysis

The amino acid sequence of γ-H was determined by Edman sequencing of the intact peptide (34 residues) and sequencing of peptides generated by proteolytic cleavage with pepsin and trypsin (Figure 1) [46]. The γ-H peptide consists of 47 amino acid residues, its calculated molecular mass is 5250 Da, and its isoelectric point is 9.77 (Table 1). The basic amino acids are unevenly distributed along the polypeptide chain, being concentrated mainly in the N- and C-terminal regions.

The amino acid sequence of ω-H was determined by Mendez et al. [47] (Figure 1). The peptide is 48 amino acid residues long, while its molecular mass deduced from the amino acid sequence is 5508.2 Da. A comparison with γ-H shows that only 19 residues are conserved and another four are conserved substitutions (Figure 1).

##### 3D Structure Analysis

The solution structure of γ-H was determined by Bruix et al. [77]. It is similar to that of γ_1_-P (Figure 2). The variation mainly concerns the loop connecting the beta1 strand to the α-helix. Three disulfide bridges are located in the hydrophobic core; they connect the α-helix to the β-sheet. Positively charged residues are located on the side of the β-sheet opposite to the α-helix [77].

The 3D structure of ω-hordothionin was studied by Bruix et al. [89]. The ω-hordothionin was shown to exist in aqueous solution as a mixture of two different isoforms, which are *cis-trans* isomers around the Phe12‒Pro13 peptide bond. The global fold of both isoforms is virtually identical. It is similar to that of γ-H. The structural variation associated with *cis-trans* isomerization is restricted to the region connecting the first β-strand with the α-helix [89].

##### Biological Activity

The γ-H was shown to inhibit translation in eukaryotic mammalian (reticulocyte and mouse liver) and nonmammalian (*Artemia* embryo) cell-free systems, as well as in the prokaryotic and four plant systems [46]. The inhibition activity in the bacterial system was lower than in eukaryotic ones (except for *H. vulgare*). The authors suggest that the peptide primarily inhibits initiation of translation and also disturbs some elongation steps. The peptide was demonstrated to promote phosphorylation of elF-2alpha. The ω-H, similarly to γ-H, inhibited translation in cell-free systems from rat liver and rabbit reticulocytes and in the prokaryotic cell-free system (*E. coli*) [47]. However, in contrast to γ-H, ω-H did not inhibit protein synthesis in plant systems, such as *T. aestivum*, *Cucumis sativus* L., *Vicia sativa* L., and *H. vulgare*.

The γ-H displayed α-amylase inhibitory activity of 40% on human saliva α-amylase [47], while ω-H showed no inhibitory effect. Both hordothionins failed to inhibit α-amylases from porcine pancreas, fungi, and bacteria [47].

##### Structure–Function Relationships

The role of disulphide bonds in translation inhibition by the γ-hordothionin was studied [46]. Disruption of disulfphide bridges by carboxymethylation of the peptide decreased the translation inhibition effect.

#### 4.2.2. Defensin-Like Peptides Identified in *H. vulgare* by In Silico Mining

We discovered 18 DEFL sequences in *H. vulgare* in the NCBI and UniProt databases (Tables S1 and S2). They form three main clusters in the phylogenetic tree based on the DEFL precursor sequences (Figure S7). Four pairs of precursors possess the same mature peptide domain (Table S3). On the phylogenetic tree constructed on the basis of the mature peptide sequences of all grass defensins, *H. vulgare* defensins do not form species-specific clusters, but are dispersed throughout the tree and group together with DEFLs from *Triticum* and *Aegilops* species, along with *B. distachyon* belonging to the Pooideae subfamily (Figure 3).

### 4.3. Sorghum

Defensins named Sialpha1, Sialpha2, and Sialpha3 (SIα1, SIα2, SIα3) from *Sorghum bicolor* (L.) Moench were first isolated and sequenced by Bloch and Richardson [50].

#### 4.3.1. Sialpha1‒3

##### Isolation

Flour from sorghum seeds was extracted with 0.1 M HCl containing 0.1 M NaCl. The supernatant was adjusted to pH 7.0 and precipitated proteins were removed by centrifugation. Ammonium sulphate was added to the supernatant to 60% saturation. Precipitated proteins were dissolved in 0.05 M tris-HCl buffer (pH 7.0) containing 0.1 M NaCl and dialyzed against the same buffer. Insoluble proteins were removed by centrifugation, while the supernatant was applied to a Red Sepharose CL-6B column. The bound proteins were eluted with 0.05 M tris-HCl (pH 7.0) containing 3M NaCl and further separated by RP-HPLC on a Vydac C18 column in a linear acetonitrile gradient containing 0.1% TFA [50].

##### Amino Acid Sequencing and Primary Structure Analysis

The amino acid sequences of SIα1, SIα2, and SIα3 were determined by automated Edman degradation of peptides obtained by proteolytic cleavage with trypsin, chymotrypsin, and thermolysin (Figure 1) [50]. The calculated molecular masses of the peptides are 5382, 5316, and 5204 Da, respectively; their main characteristics are given in Table 1. The primary structure of the peptides SIα2 and SIα3 is very similar (87% sequence identity), while SIα1 has only 42% identity with SIα2 and 38% with SIα3. Disulphide bridges were determined by a combination of Edman sequencing and mass spectrometry (MS) as follows: C3‒C47, C14‒C34, C20‒C41, C24‒C43 [90]. They occupy the same position as in γ-purothionins and hordothionins. Comparison of the amino acid sequences of two sorghum peptides SIα2 and SIα3 with wheat γ-purothionins and barley γ-hordothionin revealed high sequence similarity.

##### 3D Structure Analysis

**Figure 4:**
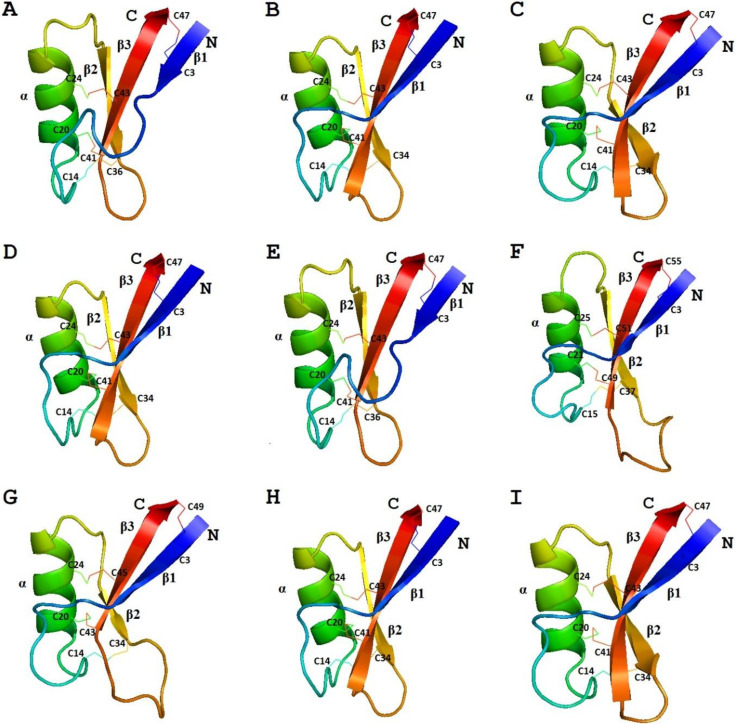
The 3D structure models of sorghum defensins (**A**) Sialpha1 and (**B**) Sialpha2 with γ-hordothionin (PDB 1GPT) and γ_1_-purothionin (PDB 1GPS) as templates, respectively; rice defensin (**C**) OsDEF8 with ZmD32 (PDB 6DMZ) as a template; maize defensins (**D**) γ_1_-zeathionin with γ_1_-purothionin (PDB 1GPS) as a template; (**E**) γ_2_-zeathionin and (**F**) ZMES1 with γ-hordothionin (PDB 1GPT) as a template; (**G**) ZmDEF1 with ZmD32 (PDB 6DMZ) as a template; lyme grass defensin (**H**) La-D4 with γ_1_-purothionin (PDB 1GPS) as a template; and barnyard grass defensin (**I**) Ec-AMP-D1 with ZmD32 (PDB 6DMZ) as a template. Modeling was carried out using SWISS-MODEL [91]. Disulfide bonds are shown by thin lines. The N-and C-termini are indicated by N and C, respectively. Secondary structure elements are denoted by α for α-helix and β for β-strand.

We carried out molecular modeling of sorghum defensins using the SWISS-MODEL program [91]. γ-H (PDB 1GPT) was used as a template for SIα1, while γ_1_-P (PDB 1GPS) was used for SIα2 and SIα3 (Figure 4) [92]. The predicted 3D structures of SIα2 and SIα3 were virtually identical, which is not surprising considering the amino acid sequences of both peptides are very similar. Therefore, only SIα2 is presented in Figure 4. The 3D structure of SIα1 differs from that of SIα2 and SIα3 in the length of beta1 and beta3 strands and the loops connecting the secondary structure elements.

##### Biological Activity

All sorghum peptides are potent inhibitors of the α-amylases from the guts of locusts and cockroaches and are weak inhibitors of the enzymes from the fungus *Aspergillus oryzae* and human saliva [50]. They also fail to inhibit the α-amylases from porcine pancreas, barley, and *Bacillus* spp. [50]. The ability of sorghum peptides to inhibit α-amylases from insects points to their involvement in defense against insect pests.

Of the three sorghum defensins, only SIα1 at high concentrations exhibited antifungal activity against *Botrytis cinerea* (IC_50_ = 100 µg/mL), *Cladosporium sphaerospermum* (IC_50_ = 80 µg/mL), and *Trichoderma viride* (IC_50_ = 50 µg/mL). The inhibitory activity against *Fusarium culmorum* and *Penicillium digitatum* was much weaker (IC_50_ > 200 µg/mL) [80].

#### 4.3.2. Defensin-Like Peptides Identified in *S. bicolor* by In Silico Mining

An in silico search for defensin-like sequences in the NCBI and UniProt databases resulted in identification of 29 sequences of DEFL peptides in sorghum (Tables S1 and S2), which form four groups of related peptides (Figure S8). Of these, six DEFLs form three pairs with identical mature peptides (Table S3). On the phylogenetic tree comprising all grass defensins, sorghum DEFLs mostly group together with maize and sugarcane sequences, reflecting the evolutionary relatedness of these species (Figure 3).

### 4.4. Rice

The following defensins of rice *Oryza sativa* L. were studied in detail: OsDEF7 and OsDEF8 by Tantong et al. [67] and Weerawanich et al. [93]; OsDEF7, named OsAFP1, was further studied by Sagehashi et al. [94] and Ochiai et al. [95], while Cal1, identical to OsDEF7, was analyzed by Luo et al. [96].

#### 4.4.1. OsDEF7 and OsDEF8

By mining Phytozome and Gramene databases, 57 genes encoding OsDEFs and OsDEF-like peptides were discovered by Tantong et al. [67]. Using in silico gene expression and coexpression network analyses, two genes, *OsDEF7* and *OsDEF8*, which coexpressed with pathogen-responsive genes, were selected for detailed studies.

##### Sequence Analysis

Amino acid sequences of OsDEF7 and OsDEF8 were deduced from the nucleotide precursor sequences (Figure 1) [67]. The predicted lengths of the signal peptides are 31 and 25 amino acids, respectively. The lengths of the mature OsDEF7 and OsDEF8 peptides are 49 and 48 residues, their calculated molecular masses are 5552 and 5350 Da, while the pI values are 8.92 and 9.12, respectively (Table 1). The sequence similarity between OsDEF7 and OsDEF8 is 69%.

##### 3D Structure Analysis

The 3D structure of OsDEF7 (OsAFP1) as a dimer was determined by X-ray crystallography (Figure 2) [97]. The fold of the peptide is typical for plant defensins harboring the CSαβ motif. Arg1, His2, Leu4, Arg9, and Phe10 were demonstrated to be important for the dimer formation [97].

According to the results of molecular modeling using the structure of either NaD1 (PDB 1MR4) or ZmD32 (PDB 6DMZ) as a template, the three-dimensional structure of OsDEF8 is similar to that of OsDEF7 [67,92]. The differences between the two peptides occur in the lengths of beta2 and beta3 strands, and in the length of the loop between these beta-strands (Figure 4). In this loop, OsDEF8 has more positively charged amino acids than OsDEF7.

##### Recombinant Production

OsDEF7 and OsDEF8 were produced as fusion proteins with GST. Rosetta-gami *E. coli* (DE3) cells were transformed with the pGEX-6P-3-OsDEF7 and -OsDEF8 plasmids. The fusion proteins were purified on a Pierce glutathione spin column. The GST fusion partner was cleaved from the purified proteins by digestion with PreScission protease [67].

##### Biological Activity

Recombinant OsDEF7 and OsDEF8 exhibited antimicrobial activity in in vitro assays against Gram-negative bacteria *Xanthomonas oryzae* pv. *oryzae*, *X. oryzae* pv. *oryzicola*, and *Erwinia carotovora* ssp. *atroseptica* [67]. For OsDEF7, the minimum inhibitory concentration (MIC) value for inhibition of both *Xanthomonas* strains was 3.9 µg/mL; for OsDEF8, the MIC value for *X. oryzae* pv. *oryzae* was the same as for OsDEF7, while for *X. oryzae* pv. *oryzicola* it was as low as 0.6 µg/mL. The MIC value for *E. carotovora* was 0.63 µg/mL. However, both peptides were inactive against Gram-positive bacteria at the concentrations tested. Antifungal assays showed that both peptides inhibited germination of *Helminthosporium oryzae* conidia at MIC = 15 µg/mL but failed to inhibit *F. oxysporum* f.sp. *cubense* spore germination at MIC > 120 µg/mL, although both peptides inhibited the hyphal elongation of both fungi.

Further studies of the antimicrobial activity of OsDEF7 and OsDEF8 in planta showed that *Nicotiana benthamiana* Domin leaves infiltrated with *OsDEF7* or *OsDEF8* constructs exhibited inhibitory activity against *X. campestis* pv. *glycines* [93].

The inhibitory activity of OsDEF7 named OsAFP1 against the rice blast pathogen *Pyricularia oryzae* was studied by Sagehashi et al. [94]. The recombinant peptide was expressed in *E. coli* as a fusion protein with GST. The recombinant OsAFP1 exhibited high antifungal activity against *P. oryzae* (IC_50_ = 0.99 µg/mL), *Rhizoctonia solani* (IC_50_ = 1.48 µg/mL), and *Gibberella fujikuroi* (IC_50_ = 3.75 µg/mL), but no antibacterial activity against *Burkholderia plantarii*, *B. glumae*, or *Acidovorax avena* ssp. *avenae* at the concentrations tested.

Ochiai et al. [95] studied the effect of OsDEF7 on the growth of human pathogenic fungi and showed that the peptide exerted fungicidal activity against *Candida albicans* at a concentration of 22.2 µg/mL. The recombinant peptide also inhibited the growth of two *Saccharomyces cerevisiae* strains (MIC = 22.2 µg/mL and 88.8 µg/mL), but failed to inhibit the growth of human pathogenic bacteria, such as Gram-negative bacteria *E. coli* K-12 and *Porphyromonas gingivalis*, and Gram-positive bacteria *Streptococcus mutans*, *Staphylococcus aureus*, and *Propionibacterium acnes* at concentrations below 177.7 µg/mL. The peptide retained activity after heating at 100 °C for 10 min and serum incubation at 37 °C for 24 h. Analysis of the mode of action of the peptide showed that it binds to phosphatidylinositols of the *C. albicans* cell membranes and induces apoptosis [95,97].

##### Structure–Function Relationships

**Figure 5:**
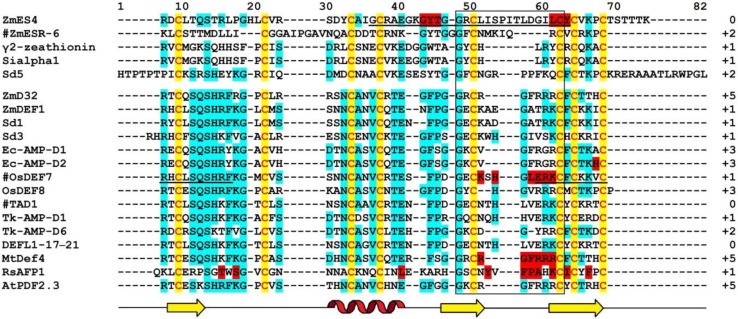
Sequence alignment of grass defensins with antifungal activity. Defensins MtDef4, RsAFP1, and AtPDF2.3 are included for comparison. Cysteine residues are shaded yellow, while identical amino acids are shaded cyan. Functionally significant residues are highlighted in red [25,79,94,95,98,99]. Regions important for antifungal activity are underlined. The position of β-strands (yellow) and the α-helix (red) are indicated below the figure. A black frame borders the γ-core. The γ-core charge values are shown on the right. The # symbol on the left indicates peptides possessing both antifungal and antibacterial activities.

Structure–function relationships in rice defensins were studied using OsDEF7 (OsAFP1) as a model [94,95]. Eight short peptides covering the entire sequence of OsDEF7 were synthesized. The inhibitory activity of the short peptides was assayed against the plant (*P. oryzae*) and human (*C. albicans*) pathogens [94,95]. Peptides 1 (residues 1‒10), 2 (residues 5‒15), 7 (residues 35‒44), and 8 (residues 40‒49) derived from the N-and C-terminal regions of OsDEF7 displayed antimicrobial activity against *P. oryzae* (IC_50_ = 0.41‒1.42 µg/mL). Peptides 3, 4, 5, and 6, corresponding to the central part of the molecule, showed no antifungal activity against this fungus. The activity of peptide 1 was higher than that of the whole peptide, while peptides 2, 7, and 8 had approximately the same activity as OsDEF7 (Figure 5).

Peptide 1 also showed pronounced antifungal activity against *C. albicans* (IC_50_ = 7.6 µg/mL); the activities of peptides 2, 7, and 8 were weaker (IC_50_ = 12‒21.9 µg/mL) than that of peptide 1, suggesting that approximately 10 amino acids from the N- and C-termini are important for activity. The role of the C-terminal region of OsDEF7 in the activity against *C. albicans* was also studied by site-directed mutagenesis. Six residues (Lys35, His 37, Leu39, Glu40, Arg41, and Lys42) were subsequently substituted for alanine in the peptide 7 region. Recombinant peptides were produced in *E. coli* and purified. All of them displayed much weaker activity, especially Leu39 and Arg41 mutants, pointing to the key role of these residues in the antifungal activity of OsDEF7 (Figure 5).

##### Expression Analysis

In silico gene expression and coexpresssion network analyses showed that OsDEF7 and OsDEF8 were coexpressed with pathogen-responsive genes [67]. Both *OsDEF7* and *OsDEF8* genes had high expression levels in all tissues examined, however *OsDEF7* was more highly expressed in all tissues. *OsDEF7* and *OsDEF8* were up-regulated by infection with the fungus *Magnaporthe grisea* and *X. oryzae* [93]. However, some pathogens down-regulated expression of these genes. Furthermore, in silico analysis showed that both genes were up-regulated by abiotic factors, such as drought, cold, dehydration, and imbibition.

In addition to in silico analysis, expression profiles of *OsDEF7* and *OsDEF8* genes in *O. sativa* ssp. *indica* and *O. sativa* ssp. *japonica* were studied by quantitative RT-PCR [93]. Up-regulation of both genes in the leaves 8 days after infection with *X. oryzae* pv. *oryzae* was confirmed.

#### 4.4.2. CAL1 (OsDEF7)

A defensin CAL1 encoded by the *CAL1* (*Cd Accumulation in Leaf*) gene was studied by Luo et al. [96]. The gene was discovered during screening of 212 rice accessions in the indica cultivar Tainan1 (TN1) hyperaccumulating Cd. Cd is a toxic heavy metal, which has no biological role in plant metabolism [100]. Cd causes diseases in plants and humans. Reducing Cd accumulation in crop plants, such as rice, is of prime importance for production of safe food.

##### Sequence Analysis

*CAL1* encodes a defensin precursor with a signal peptide and a mature peptide of 49 amino acid residues. The mature peptide sequence of CAL1 is identical to that of OsDEF7 (Figure 1).

##### Biological Activity

Luo et al. [96] demonstrated that CAL1 positively regulates Cd accumulation in rice leaves. Using in vitro metal binding assays, it was shown that both the full length and the mature CAL1 can bind Cd. It was suggested that CAL1 acts via chelation of Cd in the cytosol, facilitating its secretion to the extracellular space, lowering the cytosolic Cd concentration, and enhancing long-distance transport via xylem vessels from roots to shoots [96]. CAL1 does not influence Cd accumulation in rice grains or the accumulation of other essential metals; thus, it shows promise to remediate paddy soils and produce rice varieties with safe grains.

##### Structure–Function Relationships

Using site-directed mutagenesis, it was demonstrated that mutations of only 3 cysteine residues Cys55, Cys65, and Cys75 in CAL1 significantly decreased the Cd binding capacity of the peptide [96]. It was suggested that Cd is coordinated to three thiol groups in CAL1 to form a stable Cd:3(SH‒) complex similar to that observed in phytochelatins, low-molecular cysteine-rich proteins involved in detoxification of heavy metals. One conserved variation in the mature peptide region was discovered in CAL1—Leu70/Val. The Cd levels in the leaves and xylem sap of CAL1^L70/V^ were significantly lower than in CAL1 varieties, suggesting that Leu70/Val substitution reduces long-distance transport of Cd to aerial parts of the plant, possibly due to the disruption of the Cd binding capacity [96].

##### Expression Analysis

*CAL1* is expressed preferentially in the root exodermis and xylem parenchyma cells. *CAL1* expression in the roots is induced by Cd. Ectopic expression of the gene enhanced Cd tolerance and accumulation in yeasts and *E. coli* cells [96].

#### 4.4.3. Defensin-Like Peptides Identified in *O. sativa* by In Silico Mining

Silverstein et al. [38] discovered 93 genes encoding DEFLs in the rice genome. Of these sequences, a total of 41 sequences possessed the cysteine motif typical for classical defensins (Tables S1 and S2). Sequences of rice DEFLs are diverse (Figure S9). Only two DEFLs have the same mature peptide (Table S3). On the phylogenetic tree based on the mature defensin sequences of different grasses, nineteen rice DEFL sequences form a separate species-specific cluster (Figure 3). A smaller cluster includes 7 rice DEFLs together with maize sequences. The remaining single DEFL sequences are distributed throughout the tree.

### 4.5. Maize

Two defensins named γ_1_- and γ_2_-zeathionins (γ_1_-Z and γ_2_-Z) were isolated from *Zea mays* L. [68]. The structure of several defensins—ZmESR-6, ZmES1‒4, ZmDEF1, and ZmD32—was deduced from the cDNA nucleotide sequences, and the properties of the recombinant peptides were studied [69,70,71,72,101].

#### 4.5.1. γ-Zeathionins

##### Isolation

Isolation of maize defensins γ_1_-Z and γ_2_-Z from grain was described in detail by Castro et al. [68]. Defensins were isolated from flour by extraction with 0.1 M HCl containing 0.1 M NaCl. Peptides were precipitated by ammonium sulfate added to 60% saturation and separated on a Red Sepharose CL-6B column. The defensin-containing fractions bound to the column were eluted with 0.1 M tris-HCl (pH 7.0) containing 3.0 M NaCl and further separated by RP-HPLC on a Vydac RP-C_8_ column in a linear acetonitrile gradient containing 0.1% TFA. Defensins were purified by rechromatography on a protein and peptide RP-C_18_ column.

##### Amino Acid Sequencing and Primary Structure Analysis

Amino acid sequences of γ_1_-Z and γ_2_-Z were determined by Edman degradation of the intact peptides and confirmed by sequencing of peptides obtained by enzymatic cleavage (Figure 1) [68]. The calculated molecular masses of γ_1_- and γ_2_-zeathionins are 5199.1 and 5368.2 Da, respectively. The pI values of the peptides are 8.95 and 8.51, respectively (Table 1). The γ_1_-Z and γ_2_-Z show low sequence similarity with each other (32% sequence identity). In contrast, sequence comparison of γ_2_-Z with SIα1 shows that the peptides differ in a single amino acid residue—Asp at position 28 in γ_2_-Z is substituted for Glu in SIα1 (Figure 1).

The structure of the γ_2_-Z precursor (named PDC1) was deduced from the cDNA sequence [102]. The sequence of the mature PDC1 peptide was identical to that of maize γ_2_-Z and had the typical features of plant defensins, including a signal sequence of 35 amino acids and a characteristic defensin domain of 47 amino acids containing 8 cysteines arranged in a defensin-specific motif.

##### 3D Structure Analysis

The 3D structure of γ-zeathionins was modeled using γ_1_-P (PDB 1GPS) as a template for γ_1_-Z and γ-H (PDB 1GPT) for γ_2_-Z (Figure 4) [92]. The predicted structures of both peptides display the defensin-specific fold. The differences between the two peptides reside in the length of beta1 and beta3 strands and in the loops connecting the beta1 strand with the alpha-helix and beta2 with beta3.

##### Biological Activity

The sodium channel blocker activity of γ-zeathionins was first reported by Kushmerick et al. [53]. Using the whole-cell patch clamp technique, they showed that both γ_1_-Z and γ_2_-Z rapidly and reversibly inhibited sodium channels in the animal GH3 cell line.

The antimicrobial activity of the recombinant γ_2_-Z (PDC1) produced both in *E. coli* and *Pichia pastoris* was studied by Kant et al. [102]. The recombinant PDC1 peptide was purified on a nickel resin column and tested against *Fusarium graminearum*. Both recombinant proteins at concentrations of 5‒50 µg/mL exhibited antifungal activity against this pathogen in spore germination assays, however the protein expressed in *P. pastoris* appeared to be a more potent inhibitor of the fungal growth. The deleterious effect of the peptide on *F. graminearum* hyphae was also recorded by fluorescent microscopy after staining with fluorescein diacetate.

##### Structure–Function Relationships

The importance of Arg39 in loop 5 of γ-zeathionins for the channel-blocking activity was deduced from sequence comparisons with µ-conotoxins of *Conus geographus* L., which are structurally related peptides with 3 disulphide bridges [53]. It was established that in the toxins, Arg13, which interacts with Glu758 and Glu403 on the channel, is the most important residue for the channel-blocking activity. This residue is located in a cluster of positively charged amino acids in the C-terminal region of the molecule and is conserved among γ-thionins, suggesting similar channel-blocking activity for other members of the family.

#### 4.5.2. ZmESR-6

The structure of the ZmESR-6 precursor protein was deduced from the nucleotide sequence of a *Z. mays* cDNA clone. This defensin is expressed in the basal region of immature kernels [69].

##### Sequence Analysis

*ZmESR-6* cDNA encodes a small (11.1 kDa) protein with structural features of plant defensins. In addition to a predicted N-terminal signal peptide (27 amino acids) present in all defensin precursors, the precursor has a C-terminal acidic pro-peptide (29 amino acids long, with the predicted cleavage site between Cys-79 and Ala-80), which was also discovered in the floral defensin precursors of the Solanaceae family [32]. The mature peptide ZmESR-6 has a molecular mass of 5.5 kDa and is 52 amino acid residues long, while its pI is 8.73 (Figure 1 and Table 1). The mature peptide shares low sequence similarity with γ-purothionins (Figure 1).

##### 3D Structure Analysis

Secondary structure prediction for ZmESR-6 with the PHDsec program carried out by Balandín et al. [69] showed the presence of an α-helix in the central region of the molecule and two β-strands in the C-terminal region in the same positions as in plant defensins. Four disulphide bridges were predicted in ZmESR-6. Modeling of the ZmESR-6 3D structure with the SWISS-MODEL using γ_1_-P as a template, however, showed similarity with the template only in the last 37 amino acids of the mature ZmESR-6 [69].

##### Biological Activity

To explore the biological activity of ZmESR-6, it was produced as a recombinant protein in *E. coli* cells. The recombinant ZmESR-6 protein was tested against Gram-positive (*Clavibacter michiganensis* ssp. *sepedonicus*), Gram-negative bacteria (*Rhizobium melitini* and *Xanthomonas campestris*), and fungi (*Botrytis cinerea*, *Fusarium oxysporum* f. sp. *lycopersici*, *F. oxysporum* f. sp. *conglutinans*, and *Plectosphaerella cucumerina*). The peptide showed strong inhibitory activity (IC_50_ values from 1.1 to 82.7 μg/mL) against all tested bacterial and fungal plant pathogens [69].

##### Expression Analysis

The *ZmESR-6* transcript was detected only in immature kernels in the embryo surrounding region (ESR). At the grain filling phase, ZmESR-6 was accumulated in the placentochalaza cells. Expression was not induced by wounding or by salicylic or jasmonic acid [69].

#### 4.5.3. ZmES Defensins

Four genes named *ZmES1‒4* (*Zea mays embryo sac*) were isolated from a cDNA library of maize egg cells and analyzed by Cordts et al. [70].

##### Sequence Analysis

The *ZmES* genes encode small, cysteine-rich proteins with N-terminal signal peptides (ES1‒4). The predicted cleavage site of the signal peptide is between residues 31 and 32 in ZmES1 and between residues 30 and 31 in all other ZmES peptides. The mature peptides encoded by *ZmES2* and *ZmES3* are identical. The predicted molecular mass of the peptides is 6.5 kDa, while the pI values vary from 8.20 to 8.72 (Table 1). The peptides are highly conserved with two amino acid substitutions in the C-terminal region of the molecule. The ES4 sequence is 97% identical to that of ES2 and ES3 and 91% identical to the ES1 sequence. The sequence identity between ZmES1‒4 and other γ-thionins is 36‒41%, while the sequence similarity is 46‒53% (Figure 1) [70].

##### 3D Structure Analysis

The 3D structures of ZmES1‒4 peptides modeled with γ-H as a template were identical due to the high amino acid sequence similarity and coverage of only the first 55 amino acid residues (Figure 4) [92]. Although the mature maize peptides are longer than most grass defensins studied, their proposed tertiary structure is similar to that of plant defensins. Compared to γ-zeathionins, ZmES1‒4 peptides have longer loops between beta2 and beta3 [70,92].

##### Biological Activity

Using RNAi knockdown and a synthetic ZmES4 protein, it was found that ZmES4 induces pollen tube burst of 60.8% at 3.3 µg/mL in maize by opening the potassium channel KZM1 [103]. The effect is observed at peptide concentrations of 0.7‒3.3 µg/mL. It was suggested that ZmES4 is released from the synergid cells and subsequently interacts with KZM1, resulting in channel opening, K(+) influx, water uptake, and osmotic tube burst.

At high peptide concentrations (65‒590 µg/mL), ZmES1‒4 peptides inhibited germination of *F. graminearum* conidia and *Ustilago maydis* spores. ES1 at a concentration of 590 µg/mL caused 66.7% inhibition of *F. graminearum* conidia germination, while ES4 suppressed germination of 67.8% conidia [98]. The same peptide concentrations inhibited germination of 55.9 and 56.4% of *Ustilago* spores, respectively.

##### Structure–Function Relationships

In order to identify regions important for pollen tube burst and antifungal activity of ES peptides, the mature peptides were divided into 5 fragments, named ES-a, ES-b, ES-c, ES-d, and ES-e (13‒16 amino acids in length), covering the entire sequence. These peptides and ES-d substitution variants were produced for functional analysis [98]. Studies of the effect of ES4 and ES4-derived peptides on pollen tube burst in maize showed that the ES-d peptide encompassing the loop between Cys5 and Cys6 was even more active than the full-length peptide, showing induction of pollen tube burst of 82.5% at 0.8 µg/mL. Peptides derived from other ES regions showed low if any pollen tube burst activity. To identify amino acid residues involved in maize pollen tube burst, ES-d and its 15 mutated versions were applied to germinated maize pollen. The results showed that some mutations, such as mutations of Leu3, Ile4, or Tyr15 in ES-d suppressed pollen tube burst to less than 8%, thus pointing to their important role in the process. In contrast, mutations of Gly1, Arg3, Ala4, or Glu5 in ES-c had an opposite effect and strongly increased induction of pollen tube burst.

To study the antimicrobial effect of ES-c and ES-d on two maize pathogens, *F. graminearum* and *U. maydis*, their spores and conidia were inoculated with increasing concentrations of ES peptides. Peptide concentrations of 0.2‒0.9 µg/mL used for pollen tube burst assays had no effect on germination of conidia and spores. However, at much higher concentrations (16.8‒163.4 µg/mL) they showed dose-dependent inhibition of germination after 24 h. Similarly to the pollen tube burst activity, ES-c and ES-d peptides displayed inhibitory activity, while other peptides did not. Mutations of Gly8, Tyr9, or Thr10 in ES-c; and mutations of Lys13, Cys14, or Tyr15 in ES-d significantly increased fungal germination to 66% (Figure 5). It was also shown that ES-d and ES-c peptides bound differently to *Fusarium* and *Ustilago* cells; suggesting that they interact with different fungal targets [98]. The C-terminal fragment ES-d bound only to cell surfaces of *F. graminearum*, while the ES-c peptide bound to cell surfaces and accumulated inside the cells. In *U. maydis*, both fragments were found inside the cells. At higher concenrtrations, only ES-c accumulated in the vacuoles. Sequence comparisons of ES peptides with other members of the plant defensin family showed that the amino acid residues in ES-c and ES-d that are important for fungal growth inhibition are more conserved within plant defensins and DEFLs, while ES-c and ES-d residues that are essential for the maize pollen tube burst activity are highly conserved within the ES family [98].

##### Expression Analysis

*ZmES* genes are specifically expressed in all cells of the embryo sac and repressed after fertilization, which supported the hypothesis of their involvement in protection of the female gametophyte during fertilization [70].

#### 4.5.4. ZmDEF1

A seed-specific cDNA encoding *Z. mays* defensin 1 (*ZmDEF1*) was isolated from the maize cultivar NongDa108 using primers designed for the nucleotide sequence of *T. aestivum* defensin (AB089942) [71]. The same *ZmDEF1* gene was isolated from Maison, a Vietnamese maize cultivar highly resistant to weevils [101].

##### Sequence Analysis

The ZmDEF1 precursor contains a predicted signal peptide of 31 amino acids and a mature peptide of 49 amino acids. The calculated molecular mass of the peptide is 5.4 kDa and pI = 8.92 (Figure 1 and Table 1). The sequence identity with TAD1 amounts to 63%.

##### 3D Structure Analysis

We predicted the 3D structure of ZmDEF1 by molecular modeling using ZmD32 (PDB 6DMZ) as a template (Figure 4). When compared to γ_1_-Z and γ_2_-Z, the length of all three β-strands and the loops connecting them is different, while the global defensin-specific fold is preserved [92].

##### Recombinant Production

The recombinant ZmDEF1 was produced in *Pichia pastoris* with a 6-x His-tag at the C-terminus [71]. Transgenic tobacco lines expressing the *ZmDEF1* gene under the control of the cauliflower mosaic virus (CaMV) 35S promoter were generated by *Agrobacterium*-mediated transformation [71]. The *ZmDEF1* gene under the control of a seed-specific promoter was also expressed in tobacco seeds [101]. The same gene was transferred to two maize cultivars, LC1 and LVN99, using immature embryos to improve weevil resistance [104].

##### Biological Activity

The recombinant ZmDEF1 produced in *P. pastoris* displayed inhibitory activity against the oomycete *Phytophthora parasitica* var. *nicotianae*, both in terms of spore germination and hyphal growth [71]. Constitutive expression of the *ZmDEF1* gene under the control of the cauliflower mosaic virus (CaMV) 35S promoter in transgenic tobacco plants conferred enhanced resistance to *P. parasitica* var. *nicotianae*, as shown by the antifungal assays with detached leaves [71].

Vi et al. [101] showed that seeds obtained from the tobacco transgenic lines expressing the *ZmDEF1* gene and supplied as food to maize weevils (*Sitophilus zeamais* Motsch.) caused their death after 6 days of the experiment. The protein extracts from seeds of the transgenic plants reduced weevil α-amylase activity by 67.9–71.4% in comparison with protein extracts from nontransgenic plants [101]. Furthermore, overexpression of the *ZmDEF1* gene in two Vietnamese maize cultivars (LC1 and LVN99) resulted in increased ability of the seed protein extracts to inhibit the α-amylase activity of *S. zeamais* larvae by 54.52‒63.09% as compared to nontransgenic plants [104].

##### Expression Analysis

Expression data demonstrated that *ZmDEF1* is expressed only in immature and mature seeds. *ZmDEF1* expression could not be detected in roots, stems, leaves, flowers, and seedlings, even after treatment with methyl jasmonate or abscisic acid [71].

#### 4.5.5. ZmD32

ZmD32 was discovered by searching a database comprising 1200 plant defensin sequences [105]. It has the highest net positive charge of the molecule (+10.1 at pH 7) among plant defensins [72].

##### Sequence Analysis

ZmD32 is synthesized as a precursor protein with a signal peptide. The amino acid sequence of the peptide is shown in Figure 1. Its calculated molecular mass is 5446 Da and the predicted pI is 11.03 (Table 1). The peptide displays the highest sequence similarity with three defensins: NaD2 from *Nicotiana alata* Link & Otto, PaD2 from *Parthenium argentatum* L., and NbD6 from *N. benthamiana* (85, 87, and 87% sequence similarity, respectively) [72].

##### 3D Structure Analysis

The spatial structure of the peptide was solved by NMR spectroscopy (Figure 2) [72]. The peptide adopts a characteristic CSαβ motif with an α-helix (Arg17‒Thr26) and a triple-stranded antiparallel β-sheet (Thr2‒Gln6, Gly31‒Cys34, and Cys41‒His46). The positively charged amino acid residues are located throughout the sequence of the peptide.

##### Recombinant Production

The peptide was expressed in *P. pastoris*, purified by cation-exchange and RP-HPLC [72].

##### Biological Activity

The recombinant ZmD32 was tested in vitro against both against fungi and bacteria [72]. It inhibited growth of a panel of fungi at low concentrations (IC_50_ = 2.7‒21.8 µg/mL), including *Candida albicans*, *C. auris*, *C. glabrata*, *C. krusei*, *C. parapsilosis*, *C. tropicalis*, and *F. graminearum*. The peptide also inhibited the growth of bacteria, such as *E. coli*, *Bacillus subtilis*, *Staphylococcus aureus*, and *Pseudomonas aeruginosa*, with IC_50_ values ranging from 2.2 to 9.3 µg/mL. In contrast to most defensins, ZmD32 retained antimicrobial activity at high salt concentrations—100 mM NaCl, 5 mM MgCl_2_, or 2 mM CaCl_2_. However, the rate of microbe killing at these concentrations was lower. ZmD32 was also active against *C. albicans* biofilms. The authors suggest that the high defensin charge contributes to the antimicrobial activity at high salt concentrations.

ZmD32 was active against three human tumor cell lines; however, NaD1 was much more active than ZmD32 [72].

ZmD32 also showed minimal hemolytic activity. It lysed only 1.9% of red blood cells at the concentration of 272.3 µg/mL [72].

The authors suggest that broad-spectrum antimicrobial activity, high stability against proteases, the extreme temperatures and pH characteristic of AMPs combined with low toxicity to human cells make ZmD32 an attractive template for the development of novel antimicrobials [72].

##### Structure–Function Relationships

To understand the molecular basis of the antimicrobial activity of ZmD32 at high salt concentrations, its sequence was compared with those of defensins NaD2 from *N. alata*, PaD2 from *P. argentatum*, and NbD6 from *N. benthamiana* (net charge values at pH 7 of 4.9, 6.1, and 7.6, respectively) [72]. Only the most charged NbD6 retained antifungal activity at high salt concentrations in a similar manner to ZmD32. The solvent-accessible surface plot revealed a cationic surface with pockets of increased charge density in ZmD32. These pockets are in the region of loop 5, which in ZmD32 is rich in positively charged amino acids (RGFRRR) (Figure 5). The cationic pocket on the surface of ZmD32 is not present in NaD2 or PaD2; it is found in NbD6 but it is not as charged as in ZmD32. The authors conclude that the charge density in loop 5 is necessary but not sufficient for antifungal action in high salt medium [72].

#### 4.5.6. Defensin-Like Peptides Identified in *Z. mays* by In Silico Mining

We carried out a genome-wide analysis of maize defensins. Fifty-seven maize DEFL sequences with a characteristic 8-Cys motif were retrieved from the databases (Tables S1 and S2). Of these, 18 sequences carry the C-terminal prodomain, similarly to ZmESR-6, and thus belong to class 2 defensins. Sequence comparison shows that maize DEFLs are structurally diverse, forming 10 groups of related peptides (Figure S10). A number of maize DEFLs form clusters with identical mature peptide domains (Table S3). Some DEFLs show similarity to the defensins described above, suggesting similar functions. For 2 DEFLs, association with resistance to the fungus *Aspergillus flavus* (AOA3L6G551 and NP_001146963.1) was shown [106]. On the phylogenetic tree based on mature grass defensin sequences, maize defensins either form separate clusters or group together with closely related sorghum or even rice sequences (Figure 3).

### 4.6. Sugarcane

Six sugarcane putative defensins named Sd1‒6 were identified by BLAST search in the sugarcane (*Saccharum* spp.) SUCEST database using *Pisum sativum* L. Psd1 defensin sequence as a query [73].

#### 4.6.1. Sd Defensins

##### Sequence Analysis

Amino acid sequences of Sd defensins were deduced from cDNA sequences (Figure 1). The peptides vary in length from 47 to 71 amino acid residues and all of them have pI values ranging from 8.51 to 10.59 (Table 1). The Sd defensins share sequence similarity of 27‒61% with each other [73]. The highest similarity of 61% was observed between Sd1 and Sd3, while the lowest (27%) similarity was observed between Sd3 and Sd5. Sd defensins exhibit the highest sequence similarity with defensins from the most evolutionarily closely related species—sorghum and maize. Sd6 is 100% identical to γ2-Z from *Z. mays* and 97% to SIα1. Sd2 showes 95% identity with γ1-Z and 93% with SIα3.

##### 3D Structure Analysis

The spatial structure of Sd5 was solved by NMR spectroscopy (Figure 2) [107]. Although Sd5 shares a similar fold with other plant defensins, it has an extended and unstructured C-terminal region. To elucidate the mode of action of Sd5, the dynamic properties of the defensin were explored [107]. The peptide was shown to adopt different conformations in solution, especially upon interaction with membranes.

##### Recombinant Production

*Sd1*, *Sd3*, *Sd5*, and Sd6 cDNAs were subcloned into pET28a and expressed in *E. coli* strains BL21 (DE3) and Rosetta-gami (DE3) [73]. The attempts to subclone *Sd2* and *Sd4* were unsuccessful. The soluble N-(His)6-Sd5 was purified by affinity chromatography on a nickel nitriloacetic acid agarose column and cleaved by thrombin. The recombinant peptide was purified by RP-HPLC on a C8 column in a linear acetonitrile gradient in the presence of 0.1% TFA. Sd1, Sd3, and Sd6 were isolated from inclusion bodies with urea, and similarly to Sd5 were purified by metal affinity chromatography on Ni-NTA columns. The peptides were in-column refolded. Efficient refolding was proved by MS, circular dichroism (CD) spectroscopy, and NMR. However, the yield of Sd6 was low. The His-tags were cleaved by thrombin and the target peptides were purified by RP-HPLC.

##### Biological Activity

The antifungal activity of recombinant Sd1, Sd3, and Sd5 was assayed in vitro against three fungi: *Fusarium solani*, *Aspergillus niger*, and *Neurospora crassa* [73]. All peptides proved active against these fungi. Sd1 and Sd3 had IC_50_ values ranging from 5.4 to 20.5 µg/mL, however Sd3 displayed weaker activity against *F. solani* (IC_50_ > 117.3 µg/mL). IC_50_ values for Sd5 were higher: 79.7 µg/mL for *F. solani*, 111.5 µg/mL for *N. crassa*, and above 159.3 µg/mL for *A. niger*. Since Sd6 is 100% identical to γ2-Z, it inhibits growth of *F. graminearum* at concentrations of 5‒50 µg/mL [102].

The peptides did not exhibit antibacterial activity against *Bacillus subtilis*, *E. coli*, *Staphyllococcus aureus*, or *Kocuria zhizophila* [73].

Sd1 and Sd5 were assayed for α-amylase inhibitory activity with the enzymes from human saliva, *Aspergillus oryza*, and *Bacillus subtilis* at concentrations of ≤79.7 µg/mL. No inhibition of the tested enzymes was recorded [73].

Although sugarcane defensins were not tested for ion-channel-blocking activity, Sd6, which is 100% identical to γ2-Z, inhibits sodium channels in the mammalian system [53].

##### Expression Analysis

The genes encoding Sd defensins are expressed in different sugarcane organs. The *Sd1* is expressed in lateral buds, the *Sd2* and *Sd6* genes are expressed in the calli, *Sd3* and *Sd4* are expressed in the root apex, and *Sd5* is expressed in the developing seeds [73].

### 4.7. Lyme Grass

Lyme grass *L. arenarius* is a wild-growing plant spread in Northern Europe that exhibits extreme tolerance to high salinity. Six defensins named La-D1‒6 were isolated from seeds of *L. arenarius* [74].

#### 4.7.1. La-D Defensins

##### Isolation

Defensins were isolated from seeds according to the procedure developed for *T*. *kiharae* (see above). This included acidic extraction of flour followed by affinity, size-exclusion, and RP-HPLC [65].

##### Amino Acid Sequencing and Primary Structure Analysis

The La-D4 defensin was completely sequenced by automated Edman degradation (Figure 1), while other lyme grass defensins were N-terminally sequenced [74]. The molecular mass of La-D4 is 5094 Da and the predicted pI is 8.95 (Table 1). The peptide shows the highest sequence similarity to defensins from wheat and barley—the sequence identity with γ_1_-P and γ_2_-P is 85%. The sequence identity with γ-H is 83% (94% similarity), suggesting that *L. arenarius* peptide might also inhibit translation in a cell-free system.

##### 3D Structure Analysis

We performed modeling of the La-D4 spatial structure with γ_1_-P (PDB 1GPS) as a template (Figure 4) [74]. It is of particular interest that despite sequence variation, it shows high similarity to the 3D structures of γ_1_-Z, SIα2, and SIα3. Further studies will show whether the biological activities of these defensins are similar.

#### 4.7.2. DEFLs of *L. arenarius* Seedlings Analyzed by RNA-Seq

By transcriptome analysis of *L. arenarius* seedlings, 7 DEFLs with a cysteine motif characteristic of classical defensins were discovered (Table S1) [27]. Two of these, DEFL5-4 and DEFL5-5, differ only in the signal peptide region. Two other DEFLs (DEFL5-1 and DEFL5-2) are very similar. *L. arenarius* DEFLs occur in the same clades with DEFLs of wheat and barley also belonging to the Triticeae tribe (Figure 3). Similarly to *T. kiharae*, seed defensins of *L. arenarius* differ from those expressed in seedlings.

### 4.8. Barnyard grass

Barnyard grass *E. crus-galli* is a common weed that is widely spread throughout the world. Two defensins named Ec-AMP-D1 and Ec-AMP-D2 were isolated from seeds of this species [25].

#### 4.8.1. Isolation

Ec-AMP-D1 and Ec-AMP-D2 were isolated from barnyard grass seeds using the procedure described for *T. kiharae* (see above). This included acidic extraction of flour followed by affinity, size exclusion, and reversed-phase HPLC [25].

#### 4.8.2. Amino Acid Sequencing and Primary Structure Analysis

The amino acid sequences of Ec-AMP-D1 and Ec-AMP-D2 were determined by sequencing of the intact reduced and alkylated peptides and sequencing of peptides obtained after proteolytic cleavage with Glu-C proteinase (Figure 1) [25]. The molecular masses of the peptides are 5098 and 5170 Da, respectively, while their isoelectric point is 8.74 (Table 1). The comparison of both sequences showed that the peptides differ by a single amino acid residue at position 45; in Ec-AMP-D2 His is substituted for Ala. Barnyard grass defensins show the highest similarity with *T. kiharae* defensins, ranging from 45 to 65% for different wheat peptides, with the highest sequence similarity shown for Tk-AMP-D1.1. Sequence similarity with other cereal defensins is lower: 45% for wheat γ_2_-P and barley γ-H; and 43% for wheat γ_1_-P, sorghums SIα2 and SIα3, and maize γ_1_-Z. Most of the variation between the barnyard grass and *T. kiharae* D defensins is located in the C-terminal part of the molecule.

#### 4.8.3. 3D Structure Analysis

We carried out molecular modeling of *E. crus-galli* defensin Ec-AMP-D1 using ZmD32 (PDB 6DMZ) as a template (Figure 4) [92]. The three-dimensional structure of Ec-AMP-D1 differs from that of γ_1_-P in the length of the α-helix and loops connecting the α-helix with the beta2 strand and beta1 with the α-helix.

#### 4.8.4. Biological Activity

The antifungal activity of Ec-AMP-D1 was assayed against *F. graminearum*, *F. oxysporum*, *F. verticillioides*, and *Diplodia maydis* [25]. The peptide inhibited spore germination of *F. graminearum*, *F. verticillioides*, and *D. maydis* with IC_50_ values of 5, 8.5, and 12.5 μg/mL, respectively. The Ec-AMP-D1 was only weakly active against *F. oxysporum* (IC_50_ = 102 μg/mL). The peptide was inactive against *Colletotrichum graminicola*, *B. cinerea*, and *Helminthosporium sativum* at concentrations below 30 μg/mL. Ec-AMP-D1 had no effect on the release of *P. infestans* zoospores at concentrations below 100 μg/mL; however, inhibition of hyphal elongation was recorded (IC_50_ = 25.5 μg/mL). In addition, morphological changes in the oomycete were observed, such as lysis of hyphae and sporangia in the presence of the peptide at concentrations above 100 μg/mL. The antimicrobial activity of Ec-AMP-D2 was assayed against *F. oxysporum* and *P. infestans*. Antifungal assays showed that the activity of Ec-AMP-D2 against *F. oxysporum* was the same as for Ec-AMP-D1 (IC_50_ = 102 μg/mL). However, the activity against the oomycete *P. infestans* was lower; the IC_50_ value for inhibition of hyphal growth was 50 μg/mL. In addition, Ec-AMP-D2 did not induce morphological changes in the oomycete.

#### 4.8.5. Structure–Function Relationships

The substitution of His45 in Ec-AMP-D1 for Ala in Ec-AMP-D2 reduces the inhibition of hyphal growth and abolishes morphological changes in the oomycete *P. infestans* at high peptide concentrations (above 100 μg/mL) [25].

### 4.9. Oats

Although *Avena sativa* L. is an allohexaploid species, only twelve DEFL precursor sequences were retrieved from the databases (Tables S1 and S2). All of these belong to class 1 defensins. *A. sativa* DEFL precursors form 3 groups of related peptides with varying degree of sequence similarity (Figure S11). Two DEFL precursors, AYU75326.1 and A0A2L0U0X2, have identical mature peptides (Table S1), while DEFL A0A2L0U0S0 has a mature peptide with a single amino acid substitution compared to the above-mentioned two DEFLs. DEFLs A0A223FQC9 and A0A2L0U0E9 differ in two conserved amino acid substitutions in the mature peptide region (96% sequence identity). On the phylogenetic tree, *A. sativa* DEFLs cluster together mainly with DEFLs from species of the Pooideae subfamily (wheat and related species, *B. distachyon*, and *H. vulgare*), while some others cluster together with *O. sativa* peptides (subfamily Oryzoideae) and even sugarcane, sorghum, and maize (subfamily Panicoideae) (Figure 3). Selected oat DEFLs show sequence similarity with defensins with known functions. For example, DEFL A0A2L0U0E9 has 87% sequence similarity with γ_2_-zeathionin, which displays antifungal and ion-blocking activity. Another DEFL AYU75326.1 shows the same degree of sequence similarity with the antibacterial and antifungal rice OsDEF7. A0A223FQE1 exhibits 83% sequence similarity with the antifungal defensin Ec-AMP-D1.

### 4.10. Proso Millet and Hall’s Panicgrass

Proso millet (*Panicum miliaceum* L.) is a warm season annual grass cultivated predominantly in Asian and African countries. It is the main source of highly nutritious cereal grain with high protein content, especially for the people living in arid regions [108]. Hall’s panicgrass *(Panicum hallii* Vasey) is a wild-growing perennial grass native to North America that occupies a large geographical range with diverse climatic conditions. *P. hallii* is used as a diploid model system for C_4_ perennial Panicoid grasses with complex genomes to study C_4_ photosynthesis and stress tolerance, especially adaptation to drought [109] (Table S2).

In *P. halli*, a total of 27 DEFL precursor sequences were discovered (Table S1). Seven DEFLs have a C-terminal prodomain characteristic of class 2 defensins. Of 27 sequences, 24 form pairs of highly similar peptides (Figure S12): 7 pairs have identical mature peptides (Table S3), while 4 pairs, conversely, have identical signal peptides and mature peptides with single replacements. The occurrence of pairs of highly similar sequences points to evolutionarily recent duplications of the corresponding genes. On the phylogenetic tree, *P. halli* DEFLs group together with DEFLs of other Panicoidaeae species, including *P. miliaceum*, *Setaria* spp., *Sorghum bicolor*, and *Saccharum* spp. (Figure 3).

Despite the tetraploid nature of *P. miliaceum*, only 16 DEFL precursor sequences were found in this species by database search (Table S1). Five peptides have a predicted C-terminal prodomain. Three pairs of DEFLs are very similar, differing by 2 residues in the mature peptide region (Figure S13). One DEFL precursor AOA3L6PPJ9 has the same mature peptide as *P. halli* XP_025819879.1 (Table S4).

The functions of *Panicum* defensins remain unknown. However, sequence similarity with defensins with established functions allows us to predict the biological activities for some of them. Thus, *P. halli* DEFL A0A2T7F9B1 and *P. miliaceum* DEFL A0A3L6QKH5 share 98% similarity with the sugarcane antifungal defensin Sd1, suggesting the same function for the *Panicum* peptides. The *P. halli* DEFL XP_025800124.1 displays 96% similarity with the antifungal defensin Ec-AMP-D1 of *E. crus-galli.* Two *P. halli* DEFLs A0A2T7D4P7 and XP_025822684.1 are 98% similar to SIα3 of *S. bicolor*, which is an α-amylase inhibitor.

### 4.11. Foxtail Millet and Green Foxtail

Foxtail millet (*Setaria italica* (*L.*) P.Beauv.) and its wild ancestor species green foxtail (*Setaria viridis* (L.) P.Beauv.) belong to diploid grasses with small genomes (Table S2). *S. italica* is a staple food crop widely grown in India and China, while *S. viridis* is a weed broadly distributed throughout the world. Similarly to *P. halli*, *S. viridis* serves as a model to study C_4_ photosynthesis, resistance to abiotic stress, and for gene discovery in millets [110,111].

In foxtail millet, we discovered 30 DEFL precursor sequences, 12 of which possess the C-terminal prodomain (Table S1). Sequence comparison revealed 6 groups of related peptides (Figure S14), although sequence similarity within groups is rather low, except for two precursors RCV18919.1 and RCV18918.1 with identical predicted signal peptide regions, in which sequence similarity in the mature peptide region amounts to 92%.

Fifteen *S. italica* DEFL precursors have counterparts in *S. viridis* with identical mature peptides (Table S4), reflecting the evolutionary relatedness of the two species. In agreement with this, on the phylogenetic tree based on mature defensin sequences, *S. italica* DEFLs group together with DEFLs of *S. viridis* and other DEFLs of plants belonging to the Panicoideae subfamily (Figure 3).

In *S. viridis*, we found 22 DEFL precursor sequences, eight of which have a C-terminal prodomain in addition to the signal peptide (Table S1). For the *S. viridis* DEFL sequences form three groups of related peptides, although similarly to *S. italica*, the sequence similarity within each group is rather low (Figure S15), except for DEFLs TKW28862.1 and TKW28863.1 sharing the same signal peptide. The functions of *Setaria* defensins are unknown; however, they can be inferred from sequence similarity with defensins, whose biological activity was determined. Thus, *S. italica* DEFL K3ZYN4 shares 91% sequence similarity with the antifungal Ec-AMP-D1, while DEFL A0A4U6U435 of *S. viridis* is 94% similar to the α-amylase inhibitor SIα3. The *S. italica* DEFL K3YB96 has 88% sequence similarity with the antifungal sugarcane defensin Sd3.

### 4.12. Stiff Brome

Stiff brome (*Brachypodium distachyon* (L.) P. Beauv.) is an annual grass of the genus *Brachypodium* belonging to the tribe Brachypodieae, which occupies an intermediate position between the ancestral pooids and the recently evolved Triticeae, Bromeae, and Poaceae lineages [112]. Similarly to millets, stiff brome is regarded as a model plant for functional genomics [113,114]. We identified 13 DEFLs in the *B. distachyon* genome by database search (Tables S1 and S2, Figure S16). Two pairs of DEFL sequences—A0A0Q3GLW6 and I1IW23, A0A0Q3GLW6 and A0A0Q3E274—share 96% and 89% sequence similarity, respectively. Other sequences have diverged considerably. The phylogenetic tree based on predicted *B. distachyon* mature defensins and those of other grasses disclosed similarity with DEFLs of *Triticum* species, *H. vulgare*, and *A. sativa*, *L. arenarius*; and more distantly related *O. sativa*, *P. miliaceum*, and *Z. mays* (Figure 3). For example, the sequence similarity between *B. distachyon* XP_003573541 and *P. miliaceum* A0A3L6RMQ6 amounts to 89%. The sequence similarity between *B. distachyon* DEFL I1IBT1 and *A. sativa* DEFL AYU75326.1 and the antimicrobial *O. sativa* DEFL OsDEF7 are 96% and 90%, respecetively. The sequence similarity between *B. distachyon* A0A0Q3LKK6 and *T. kiharae* DEFL1-16 is 90%. It is worth noting that *B. distachyon* DEFL A0A0Q3LKK6 has a RGFRRR motif in the γ-core region, which determines the antifungal activity of plant defensins (see below).

## 5. Molecular Diversity and Functions of Grass Defensins: The Summary

Bioinformatics-based analysis of the abundance of DEFLs in the species of the Poaceae family belonging to two clades (BOP and PACMAD), including three subfamilies (Pooideae and Oryzoideae of the BOP clade and Panicoideae of the PACMAD clade) and five tribes (Triticeae, Avenaeae, Brachypodeae, Oryzeae, Paniceae, Andropogoneae), is shown in Table S2. The examined species differ in genome size and ploidy levels. The number of DEFLs discovered by in silico mining is species-specific. *Z. mays*, *S. bicolor*, *O. sativa*, and polyploid wheat species are especially rich in DEFL sequences, while *A. sativa* and *B. distachyon* have less. As mentioned above for the polyploid wheat species and wheat subgenome donors, there is a positive correlation between the DEFL number and ploidy level. However, for the two *Panicum* species this does not hold true (Table S2).

Similarly to defensins from other plant families, grass defensins are synthesized as precursor proteins consisting of a signal peptide and a mature peptide (class 1 defensins); some precursors also contain a C-terminal prodomain (class 2 defensins). The latter occur in the Panicoideae subfamily plants and in rice (subfamily Oryzoideae). Note that earlier class 2 defensins were reported only in flowers of the Solanaceae plants and in maize (ZmESR-6) [32,69]. A characteristic feature of the signal peptide sequences of grass DEFL precursors is the presence of conserved hydrophobic polyleucine or leucine and valine motifs possibly involved in defensin trafficking and secretion to the apoplast.

Sequence comparison of grass defensins allows us to speculate on the evolution of this gene family. In each species, DEFL precursor sequences are presented by groups (subfamilies) of related peptides originating from common ancestors. Within these subfamilies, virtually identical pairs of sequences (paralogs) occur, which points to recent gene duplications as an important scenario of defensin gene evolution (Figure 3). The topic of future research is to examine whether these DEFLs diverged functionally. Sequence comparison between species demonstrates that some DEFL subfamilies form species-specific clusters (e.g., rice, *Triticum* and *Aegilops* species) (Figure 3), while some show high sequence similarity with DEFLs from closely related species (of the same genera, e.g., *Panicum* spp. or *Setaria* spp.; or tribe, e.g., Triticeae or Andropogoneae) and are not found in more evolutionarily distant species. However, some DEFLs are more conserved: highly similar orthologs are found in species belonging to different subfamilies of one or even different clades (BOP or PACMAD) (Figure 3), which means that they originated in the common ancestor before the divergence of these taxa. Note that the divergence of the BOP clade was estimated to take place at approximately 50 Ma, while divergence of the PACMAD clade occured at approximately 40 Ma [115]. We believe that these conserved DEFLs play pivotal roles in plant physiology, and therefore are of particular interest for future functional studies. In accordance with this hypothesis is the observation that the potent maize defensin ZmD32 has highly similar orthologs not only in maize, sugarcane, and *Panicum* species, but also in *T. kiharae*, *T. durum*, *A. tauschii*, and *B. distachyon* (Figure 3).

Despite sequence variation, the grass defensins studied so far share similar properties: a positive net charge of the molecule (with few exceptions) (Table 1), uneven distribution of positively charged residues concentrated mainly in the N- and C-terminal regions (Figure 1), positively charged or neutral γ-core regions (Table 1), and a similar three-dimensional structure harboring a cysteine-stabilized alpha-beta motif present in all plant defensins (Figure 2). The differences between defensins reside in the length of the secondary structure elements (α-helix and β-strands) and of the loops connecting them (Figure 2 and Figure 3). The three-dimensional structure is suggested to determine the defense properties of defensins and other AMPs [116], while sequence variation is supposed to contribute to their functional diversity [35].

Recent research described in the previous section expanded our knowledge on the functions of grass defensins. Early studies showed that they lack substantial antimicrobial activity [80,117]. However, the antimicrobial activity was later discovered in earlier isolated peptides and in novel members of the family (see above) (Figure 5). The antimicrobial activity of grass defensins was revealed by in vitro assays and by analysis of transgenic plants expressing defensin genes. However, it should be taken into consideration that the peptide concentrations used in assays by different research teams differ considerably, and the fungal or bacterial strains tested also differ. For example, in our antifungal assays of *T. kiharae* seed defensins against wheat and maize fungal pathogens, the concentrations used did not exceed 30 µg/mL. At these concentrations, Tk-AMP-D1 and Tk-AMP-D6 were weakly active [25,83]. In contrast, the maize defensin ZmES4 shown active against *Fusarium graminearum* and *Ustilago maydis* was tested at a much higher concentration (590 µg/mL) [98]. Thus, the antifungal potency of grass defensins may differ considerably. Of particular interest is that some defensins display both antifungal and antibacterial activity (wheat TAD1, rice OsDEF7, and maize ZmD32). Furthermore, some of them (OsDEF7 and ZmD32) were shown to be active not only against plant pathogens, but also against human pathogens posing a serious threat to human health, such as *Candida* spp. Of the cereal defensins studied in detail, a unique maize defensin ZmD32 retained activity at high (physiological) salt concentrations when other plant defensins were inactive, making ZmD32 a promising molecule for drug design.

The sequence comparison of antimicrobial cereal defensins clearly demonstrates that they fall into two groups. The smaller and more diverse group includes maize defensins ZmES4, ZmESR-6, γ_2_-zeathionin, and its closest relative SIα1 from sorghum and sugarcane Sd5 (Figure 5). The larger group consists of the maize ZmD32 and ZmDEF1, two defensins of sugarcane (Sd3 and Sd5), rice OsDEF7 and OsDEF8, *E. crus-galli* Ec-AMP-D1 and Ec-AMP-D2, and four defensins of wheat (TAD1, Tk-AMP-D1, Tk-AMP-D6, and DEFL1-17) (Figure 5). It is of particular interest that the antifungal defensin of *Medicago truncatula* Gaertn. MtDef4 shares high sequence similarity with cereal group 2 antifungal defensins (e.g., 79% identity and 85% similarity with ZmD32) [99], while antifungal defensin RsAFP1 displays low sequence similarity with the defensins from grasses (Figure 5) [79]. Sequence similarity within group 2 is higher than in group 1, especially in the N-terminal regions of the molecules (32 residues). Until now, the roles of particular regions of the molecule and amino acid residues in the antifungal activity have been explored only for two cereal defensins, ZmES4 from maize and OsDEF7 from rice. Beyond the grass family, the structure‒function relationships have been studied for radish (*Raphanus sativus* L., Brassicaceae family), *Medicago sativa*, and *M. truncatula* (Fabaceae family) defensins [118]. In the rice OsDEF7 defensin, the antifungal determinants are located in the N- and C-terminal regions of the molecule (see above) (Figure 5). As mentioned above, the N-terminal region of group 2 grass antifungal defensins is highly conserved, while the C-terminal region is less conserved (Figure 5). The sequence of ten C-terminal amino acid residues of OsDEF7, which shows antifungal activity, is found also in ZmDEF1 and Sd1 with two substitutions E/T and V/I. Similar sequences are found in TAD1, DEFL1-17, and Tk-AMP-D1, suggesting that these defensins may have a similar mode of antifungal action to OsDEF7. The residues in the C-terminal region of OsDEF7, which are important for the antifungal activity, are conserved in some but not all cereal antifungal group 2 defensins, and are not conserved in group 1 defensins (Figure 5). In ZmES4, the antifungal activity was shown to reside in the C-terminal half of the molecule. The residues G31, Y32, T33, L48, C49, and Y50 were identified as being vital for the antifungal activity (Figure 5). A comparison of the grass antifungal defensin sequences demonstrates that the motif GY/W/FT/S is found in all group 1 cereal defensins and in *E. crus-galli* defensins, suggesting that it may be their common antifungal determinant, while the LCY motif is specific to ZmES4.

The γ-core region, defined as GXCX_3–9_C and discovered in all antimicrobial peptides, is supposed to be crucial for the antimicrobial activity [99,119]. Some authors assume that sequence X_3–9_ within the γ-core corresponding to loop 5 is actually essential for the biological activity of plant defensins [35,41]. Comparison of amino acid sequences of antifungal grass defensins shows that the γ-core regions are variable in both sequence and length. However, at position 37, a hydrophobic residue is present in all group 1 defensin sequences, as well as a basic residue at position 38 (the numbering is according to ZmD32) (Figure 5). It deserves special attention that the γ-core sequence RGFRRR of ZmD32 is identical to that of MtDef4. For MtDef4, it was shown that peptide RGFRRR alone is capable of inhibiting fungal growth and determines the defensin morphogenicity (MtDef4 is nonmorphogenic) [99]. Subsequent studies showed that RGFRRR peptide is a translocation signal necessary for entrance into the fungal cells to act on intracellular targets [120]. Genome-wide analysis of grass defensins allowed us to discover 11 more sequences possessing the same motif in *T. kiharae*, *T. aestivum*, *T. durum*, *T. urartu*, *A. tauschii*, *L. arenarius*, maize, sugarcane, rice, *Panicum* species, and *B. distachyon* (Figure S17). A highly similar γ-core sequence HGVRRR is found in OsDEF8. Beyond the Poaceae family, the RGFRRR motif is found in the antifungal and ion-channel-blocking defensin AtPDF2.3 from *Arabidopsis thaliana* (L.) Heynh. (see below) and antifungal *Phaseolus vulgaris* L. defensin PvD2 [42,54]. Thus, it seems that this motif is present in the γ-core regions of antifungal defensins of plants belonging to different families.

The cationic residue R38 and the hydrophobic residue F37 in the γ-core of MtDef4 were shown to contribute significantly to the antifungal activity of the MtDef4 defensin from *M. truncatula* [99], and as mentioned above these residues are conserved in all group 1 antifungal cereal defensins. Studies of the mode of action of MtDef4 and its γ-core peptide pointed to the permeabilization of fungal membranes as the main mechanism involved. The same mechanism possibly operates in the case of the maize defensin ZmD32 and all other defensins possessing the same γ-core sequence. The charges of the γ-core regions in all other antifungal grass defensins vary from 0 to +3 (Figure 5). For comparison, the γ-core charge of ZmD32 is +5. Lower charge is due to the substitution of a positively charged R for a neutral residue or even a negatively charged E (Figure 5). The highest values of +3 and +5 were observed in OsDEF8, *E. crus-galli* defensins, and ZmD32. The high charge of the γ-core correlates with potent antimicrobial activity of these peptides. However, in TAD1, the γ-core charge is 0, although this defensin displays a rather strong inhibitory effect on the fungus *T. ishikariensis* and the bacterium *P. cichorii*, providing evidence that the antifungal activity of TAD1 and structurally related defensins relies on some other determinants [66,85]. Thus, a positive γ-core charge is not an obligatory requirement for the antifungal activity of grass defensins. It seems most likely that the determinants of antifungal activity are different for different grass defensins that may be associated with diverse modes of action or different molecular targets, as was demonstrated for defensins from nongrass species [118]. However, the exact molecular mechanisms underlying the antifungal activity of grass defensins remain to be elucidated.

The determinants of antibacterial activity in plant defensins are largely unknown. For nonplant defensins, the crucial roles of net positive charge, hydrophobicity, and flexibility in the antibacterial activity were postulated [121]. Analyzing plant defensins with antibacterial activity Sathoff and Samac pointed to several structural features that are inherent to AMPs with antibacterial activity [44]; among them were the ability to form oligomeric structures, the abundance of glycine residues, and the lipid-binding activity. The ability to form dimers was demonstrated for rice OsDEF7 and OsDEF8, a high percentage of glycine residues is observed in the maize ZmESR-6 (7 Gly residues) [69], and the lipid-binding activity was detected in ZmD32 [72]; all of these peptides display antibacterial activity. Analysis of the antibacterial activity of MtDef5 from *M. truncatula* using site-directed mutagenesis pointed to the importance of positively charged residues of the γ-core in the antibacterial activity of the peptide [122]. However, not all cereal defensins possessing antibacterial activity have a positively charged γ-core (e.g., TAD1) (Figure 5). This indicates that similarly to the antifungal activity, the antibacterial activity in different grass defensins is possibly realized via diverse mechanisms.

Genome-wide analysis of cereal defensins allowed us to predict defensins with antimicrobial activity in Poaceae species based on sequence similarity with the antifungal and antibacterial defensins described above (Figure 3). Thus, the antimicrobial TAD1 from *T. aestivum* has close relatives in *T. kiharae*—DEFL1-17 (98% identity) and DEFL1-5 (96% identity, 98% similarity). DEFLs 1-23, -31, -3, -45, and -48 are also very similar to TAD1. For DEFL1-48, for example, the sequence similarity with TAD1 is 90% and the sequence identity is 89%. *A. tauschii* defensin (R7W5W7) also belongs to this group of TAD1-related peptides. The antimicrobial salt-tolerant maize defensin ZmD32 has homologues in maize (A0A317Y7J20) (98% identity) and other species, such as *T. kiharae*, *T. durum*, and *B. distachyon* (Figure 3), with amino acid sequence identities varying from 81 to 87%, and the net charge of +9 or even +10, as in ZmD32. The antifungal and antibacterial rice defensin OsDEF7 has homologues in *A. sativa* (AYU75326.1 and A0A2L0U0S0, 88 and 86% similarity, respectively) and *B. distachyon* (I1IBT1, 92% similarity). Antifungal *E. crus-galli* defensins Ec-AMP-D1 and Ec-AMP-D2 have related peptides in *S. italica* (K3ZYN4, 94% similarity), *P. halli* (XP_025800124.1, 94% similarity) and *A. sativa* (A0A223FQE1, 85% identity). The antifungal sugarcane Sd3 defensin has a homologue in *S. italica* (K3YB96), with 5 nonconserved substitutions and related peptides in other species, such as *H. vulgare* (KAE8814413.1), *T. kiharae*, *A. tauschii*, *T. durum*, *Panicum* spp., and *O. sativa* (Figure 3). Thus, genome-wide analysis expands the pool of defensins for further functional studies and practical applications.

The antimicrobial activity of grass defensins revealed by in vitro assays points to their protective roles in planta. Analysis of transgenic plants expressing defensin genes confirms this hypothesis. Transgenic plants overexpressing the maize ZmDEF1 gene in tobacco conferred enhanced resistance to *Phytophothora parasitica* var. *nicotiana*, while overexpression of this gene in two Vietnamese maize cultivars LC1 and LVN99 resulted in an increased inhibitory activity against α-amylases of *Sitophilus zeamais* larvae, suggesting a role in pest defense [101,104]. Thus, the antimicrobial activity of defensins from grasses may have practical applications in crop protection to combat fungal and bacterial pathogens and insect pests, as well as in medicine.

**Figure 6:**
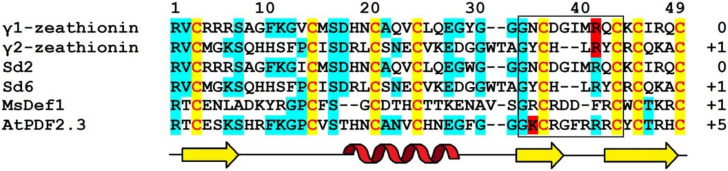
Sequence alignment of grass defensins with ion-channel blocking activity. MsDef1 and AtPDF2.3 defensins are included for comparison. Cysteine residues are shaded yellow, while identical amino acids are shaded cyan. Functionally significant residues are highlighted in red [53,54]. The position of β-strands (yellow) and the α-helix (red) are indicated below the figure. A black frame borders the γ-core. The γ-core charge values are shown on the right.

In addition to the antimicrobial activity, the Poaceae defensins were shown to display ion-blocking activity (Figure 6). Ion channel blockers are supposed to be targets for the development of novel drugs to treat human diseases including cancer, meaning grass defensins that exhibit ion-blocking activity can, thus, be used in this field. Ion-channel-blocking activity was shown for two maize defensins, γ_1_-Z and γ_2_-Z, which inhibit sodium channels [53], although they show low sequence similarity with each other. Sugarcane defensins Sd6 and Sd2 possess the same activity, since Sd6 is identical to γ_2_-Z and Sd2 has two conserved substitutions as compared to γ_1_-Z. Other examples of ion blockers among nongrass species include AtPDF2.3 from *A. thaliana*, which has been shown to inhibit potassium channels; and MsDef1 from *Medicago sativa*, which blocks L-type Ca^2+^ channels [52,54]. Both of these peptides display antifungal activity. Of the grass defensins capable of blocking ion channels, the γ_2_-Z was shown to be antifungal. Arg39 was claimed to be important for inhibition of sodium channels by maize γ_1_-Z and γ_2_-Z [53]. It is of interest that this residue is conserved in AtPDF2.3 and most antifungal defensins from grasses (Figure 5). However, it is still to be investigated whether the ion-blocking activity is related to the antifungal activity of the maize defensin γ_2_-Z. For MsDef1, it was shown that Arg38 is important for the antifungal activity. Furthermore, it was suggested that the ion-blocking activity and antifungal activity of the peptide are linked—the peptide was supposed to target fungal Ca^2+^ channels [52]. For AtPDF2.3 from *A. thaliana*, Lys33 was shown to be vital for inhibition of potassium channels; furthermore, the substitution of Gly36 for Asn greatly enhanced the ion-channel blocking activity [54]. In contrast to MsDef1, for the *A. thaliana* defensin AtPDF2.3, it was shown that the antifungal activity and the channel-blocking activity are not related [54]. Interestingly, the motif Lys-C_5_-X-Gly, similarly to that found in AtPDF2.3, is present in the antifungal defensins Tk-AMP-D6 and both *E. crus-galli* defensins (Figure 5). It remains to be studied whether these defensins possess the potassium-blocking activity.

Genome-wide analysis revealed close relatives of maize γ_1_-Z and sugarcane Sd2 in sorghum (SIα3 and SIα2), *P. halli* (A0A2T7D4P and XP_025822684.1), and *Setaria* species (A0A4U6U435_SETVI and K3YLP8_SETIT) (Figure 3). All of them possess Arg38 necessary for the ion-channel blocking activity. The γ_2_-Z and sugarcane Sd6 have homologous peptides with Arg38 in sorghum (Sialpha1) and *A. sativa* (A0A223FQC9 and A0A2L0U0E9) (Figure 3).

**Figure 7:**
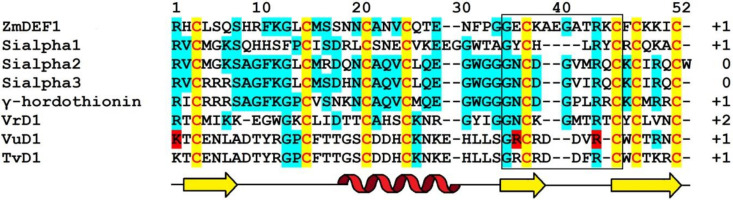
Sequence alignment of grass defensins with α-amylase inhibitory activity. VrD1, VuD1, and TvD1 defensins are included for comparison. Cysteine residues are shaded yellow, while identical amino acids are shaded cyan. Functionally significant residues are highlighted in red [51]. The position of β-strands (yellow) and the α-helix (red) are indicated below the figure. A black frame borders the γ-core. The γ-core charge values are shown on the right.

Of the grass defensins, three SIα1‒3 defensins of *Sorghum bicolor* and one defensin ZmDEF1 of *Z. mays* exhibit inhibitory activity against insect α-amylases, while the γ-hordothionin of *H. vulgare* inhibits the α-amylase from human saliva (Figure 7) [47,50,101,104]. The residues responsible for the enzyme inhibitory activity are largely unknown. Of nongrass defensins, VrD1 from *Vigna radiata* (L.) R.Wilczek, VuD1 from *Vigna unguiculata* (L.) Walp., and TvD1 from *Tephrosia villosa* (L.) Pers. were shown to inhibit α-amylases from bruchids [51,123,124]. For *Vigna* defensins, the importance of the loop between beta2 and beta3 for insect α-amylase inhibition was postulated. The introduction of loop 3 of VrD1 into the VrD2, which is inactive against *Tenebrio molitor* amylase (TMA), rendered the defensin active against the enzyme [125]. Loop 3 is the proposed region that inserts into the catalytic site of the insect α-amylase. The catalytic residues Asp185, Glu222, Asp287, and Asp94 in the cleft of TMA are supposed to interact with positively charged residues in loop 3 of the defensin. It was suggested that a short loop 3 (4 residues) and the presence of positively charged residues in this loop are necessary for inhibition [123]. However, this does not appear to be true for all α-amylase inhibitors. Thus, loop 3 in *Z. mays* ZmDEF1, which is an inhibitor of maize weevil *Sitophilus zeamais*, is long (6 residues). Conversely, inactive VrD2 has a short loop3. A conserved Arg residue at position 41 (the numbering is according to ZmDEF1), which interacts with the negatively charged residues of the active site of the insect enzyme, is present in most sequences, and is believed to be important for the ion-channel blocking activity (see above). Another conserved residue in this loop is Gly at position 38 (the numbering is according to ZmDEF1). Most cereal inhibitors carry a single acidic residue in loop 3. Two negatively charged residues Asp37 and Asp38 were supposed to prevent inactive VrD2 from interacting with the catalytic site of the enzyme that renders VrD2 inactive against the enzyme. For VuD1, the N-terminal Lys1 was shown to bind with the active sites of weevil enzymes. Some other residues, such as Arg34, Arg40, and Cys47, were also suggested to interact with the *Zabrotes subfasciatus* amylase (ZSA). A positively charged residue at position 1 is well conserved among cereal inhibitors, while at position 34 other residues are found; furthermore, Arg 34 and Arg 40 occur in noninhibitors.

In silico mining of homologues of sorghum α-amylase inhibitors SIα1‒3 revealed ion-channel blockers γ_1_-Z and γ_2_-Z from maize; sugarcane Sd2 and Sd6; and related peptides from *Setaria* spp., *P. halli*, and *A. sativa* (see above), suggesting that these grass defensins may possess two or even three functions: ion-channel blocking, α-amylase inhibition, and antifungal activity.

Inhibition of protein synthesis was shown for two barley defensins that share rather low sequence similarity. Analysis of the databases allowed us to discover another peptide (AAB01671.1) from barley with 100% similarity to ω-hordothionin. Related peptides with high sequence similarity to barley defensins were found in *T. aestivum* (AIA66988.1, AIA67001.1, 96% similarity), *T. kiharae* (DEFL1-11, DEFL1-32, 94% similarity), *Aegilops tauschii* (R7W8W0, 94% similarity), and *Avena sativa* (A0A2L0U0E7, 96% similarity). A set of peptides from *T. durum* (γ-purothionins), *T. kiharae* (DEFLs 1-12, -41, and -43, 96% similarity), *A. tauschii* (R7W7L2, 98% similarity), *L. arenarius* (La-D4, 94% similarity), and *A. sativa* (A0A286L6T1, 81% similarity) share high sequence similarity with barley γ-hordothionin.

An interesting finding of recent years regarding cereal defensin functions is the discovery that the rice defensin CAL1 is involved in Cd^2+^ tolerance in rice [96]. Cadmium is toxic for plants, animals, and humans. In plants, Cd induces oxidative stress by inhibition of antioxidative enzymes [126]. In humans, cadmium poisoning affects the liver and kidneys and induces cancer. It was suggested that the rice defensin CAL1 acts via chelation of Cd in the cytosol and promotion of Cd^2+^ long-distance root-to-shoot transport [96]. An important issue is that it does not trigger Cd accumulation in the grain. Thus, the rice defensin Ca1 can be used in phyto-remediation of polluted soils. To the best of our knowledge, this is the first report on the role of defensins in Cd tolerance in plants. Previously characterized defensins of Zn-hyperaccumulating *Arabidopsis halleri* L. were shown to be involved in Zn tolerance [60].

The abovementioned functions of grass defensins are related to stress tolerance and can be utilized to improve resistance to pathogenic microorganisms, insect pests, and heavy metals. The involvement in nondefense functions was also shown for some members of the grass defensin family. The participation of maize defensin ZmES4 in pollen tube burst was convincingly demonstrated and the mechanisms involved were suggested [98]. They include secretion of the ZmES4 defensin from the synergid cells and interaction with the KZM1 ion channel, followed by channel opening and K(+) influx, water uptake, and osmotic tube burst. The interaction of ZmES4 with the channel was found to be species-specific and supposed to represent a pre-zygotic hybridization barrier providing reproductive isolation [98].

## 6. Conclusions

Despite considerable progress in the study of defensins from grasses, only a handful of peptides of the DEFL genomic pool have been functionally characterized so far. It is becoming evident that similarly to defensins from plants belonging to other plant families, grass defensins display promiscuity—the same molecule may have more than one function (a phenomenon called pure promiscuity) or different members of the grass defensin family may exert different functions (the so-called family promiscuity) [127]. We are now beginning to understand the molecular bases of the antifungal activity of single plant defensins outside the Poaceae family [35,118]. For the grass defensins, the exact molecular mechanisms underlying different biological activities and the relationships between them are still to be investigated. Detailed functional studies of novel members of the grass defensin family (including weeds) by whole-genome analysis will help understand the extremely high adaptability of grasses and provide novel potent molecules for practical use in medicine and agriculture.

## Figures and Tables

**Table 1:** The main characteristics of isolated grass defensins.

Peptide Name	Accession Number	Peptide Length	Isoelectric Point ^1^	Net Charge at pH 7 ^1^	Prediction of Antimicrobial Activity ^2^	γ-Core Sequence	γ-Core Charge ^1^	Reference
**Wheat**								
γ_1_-Purothionin	P20158	47	9.49	+8	AMP	GNCDGPFRRC	+1	[61]
γ_2_-Purothionin	P20159	47	9.12	+6	AMP	GNCDGPFRRC	+1	[61]
TAD1	BAC10287.1	49	8.73	+4	AMP	GECNTHLVERKC	0	[66]
Tk-AMP-D1	P84963.1	49	8.21	+2	AMP	GQCNQHHVERKC	+1	[62]
Tk-AMP-D1.1	P84965.1	47	6.42	−1	AMP	GKCVGVQRHC	+2	[62]
Tk-AMP-D2	P84968.1	49	8.51	+3	AMP	GQCNQHHVERKC	+1	[62]
Tk-AMP-D3	P84970.1	45	7.03	0	AMP	GKCDGIHC	0	[62]
Tk-AMP-D4	P84971.1	45	8.51	+3	AMP	GKCDHRRC	+2	[62]
Tk-AMP-D5	P84966.1	46	8.50	+3	AMP	GKCDGYRRC	+2	[62]
Tk-AMP-D6	P84967.1	46	8.21	+2	AMP	GKCDGYRRC	+2	[62]
Tk-AMP-D6.1	P84969.1	46	8.21	+2	AMP	GKCDGYRRC	+2	[62]
Tm-AMP-D1.2	P84964.1	49	8.51	+3	AMP	GQCNQHHVERKC	+1	[62]
**Barley**								
γ-Hordothionin	P20230.1	47	9.77	+9	AMP	GNCDGPLRRC	+1	[46]
ω-Hordothionin	-	48	8.75	+4	AMP	GYCHLRRC	+2	[47]
**Sorghum**								
Sialpha1	P21923.2	47	8.51	+3	AMP	GYCHLRYC	+1	[50]
Sialpha2	P21924.1	48	8.75	+4	AMP	GNCDGVMRQC	0	[50]
Sialpha3	P21925.2	47	8.97	+5	AMP	GNCDGVIRQC	0	[50]
**Rice**								
OsDEF7	BAF09407.1	49	8.92	+5	AMP	GECKSHGLERKC	+1	[67]
OsDEF8	BAF10767.1	48	9.12	+6	AMP	GYCHGVRRRC	+3	[67]
**Maize**								
γ_1_-Zeathionin	P81008.1	47	8.95	+5	AMP	GNCDGIMRQC	0	[68]
γ_2_-Zeathionin	P81009.1	47	8.51	+3	AMP	GYCHLRYC	+1	[68]
ZmESR-6	NP_001105777.1	52	8.73	+4	AMP	GFCNMKIQRC	+2	[69]
ZmES1	AAK08132.1	61	8.50	+3	AMP	GRCLISPIPLDGILC	0	[70]
ZmES2	AAK08133.1	61	8.20	+2	AMP	GRCLISPIPLDGILC	0	[70]
ZmES3	AAK08134.1	61	8.20	+2	AMP	GRCLISPIPLDGILC	0	[70]
ZmES4	AAK08135.1	61	8.72	+4	AMP	GRCLISPITLDGILC	0	[70]
ZmDEF1	AEG19551.1	49	8.92	+5	AMP	GECKAEGATRKC	+1	[71]
ZmD32	6DMZ_A	47	11.03	+10	AMP	GRCRGFRRRC	+5	[72]
**Sugarcane**								
Sd1	CA112870.1	49	8.91	+5	AMP	GECKADGATRKC	+1	[73]
Sd2	CA095771.1	47	8.97	+5	AMP	GNCDGIMRQC	0	[73]
Sd3	CA259771.1	51	8.92	+5	AMP	GECKWHGIVSKC	+1	[73]
Sd4	CA259589.1	49	10.59	+10	AMP	GKCRGFRRRC	+5	[73]
Sd5	CA297803.1	71	9.05	+6	AMP	GFCNGRPPFKQC	+2	[73]
Sd6	CA188998.1	47	8.51	+3	AMP	GYCHLRYC	+1	[73]
**Lyme grass**								
La-D4	-	47	8.95	+5	AMP	GNCDGPLRRC	+1	[74]
**Barnyard grass**								
Ec-AMP-D1	P86518.1	47	8.74	+4	AMP	GKCVGFRGRC	+3	[25]
Ec-AMP-D2	P86519.1	47	8.74	+4	AMP	GKCVGFRGRC	+3	[25]

^1^ The isoelectric point (pI) and net charge at pH 7 were calculated by ProtParam [75]. ^2^ The antimicrobial activity of defensins was predicted using the CAMPR3 program [76].

## Supplementary Materials

The following are available online at https://www.mdpi.com/2218-273X/10/7/1029/s1, Figure S1: Multiple sequence alignment of *T. kiharae* and *T. monococcum* DEFL mature peptides, Figure S2: A phylogenetic tree based on *T. aestivum* DEFL precursor sequences, Figure S3: A phylogenetic tree based on *T. turgidum* DEFL precursor sequences, Figure S4: A phylogenetic tree based on *T. urartu* DEFL precursor sequences, Figure S5: A phylogenetic tree based on *A. tauschii* spp. *strangulata* DEFL precursor sequences, Figure S6: A phylogenetic tree based on *A. tauschii* spp. *tauschii* DEFL precursor sequences, Figure S7: A phylogenetic tree based on *H. vulgare* DEFL precursor sequences, Figure S8: A phylogenetic tree based on *S. bicolor* DEFL precursor sequences, Figure S9: A phylogenetic tree based on *O. sativa* DEFL precursor sequences, Figure S10: A phylogenetic tree based on *Z. mays* DEFL precursor sequences, Figure S11: A phylogenetic tree based on *A. sativa* DEFL precursor sequences, Figure S12: A phylogenetic tree based on *P. hallii* DEFL precursor sequences, Figure S13: A phylogenetic tree based on *P. miliaceum* DEFL precursor sequences, Figure S14: A phylogenetic tree based on *S. italica* DEFL precursor sequences, Figure S15: A phylogenetic tree based on *S. viridis* DEFL precursor sequences, Figure S16: A phylogenetic tree based on *B. distachyon* DEFL precursor sequences, Figure S17: Sequences of grass defensins with the RGFRRR motif, Table S1: Accession numbers of grass DEFL sequences, Table S2: Genome characteristics and DEFL number identified by in silico mining in grass species, Table S3: DEFLs of one species with identical mature peptides, Table S4: DEFLs of different species with identical mature peptides. References [128,129,130,131,132,133,134,135,136,137,138,139,140] are cited in the Supplementary Materials.
