# Supplementary material for: Defensins of Grasses: A Systematic Review
Source: Biomolecules. 2020 Jul 10;10(7):1029. doi: 10.3390/biom10071029 (PMC7407236; doi:10.3390/biom10071029)
Supplement: Supplementary file 1 [file biomolecules-10-01029-s001.zip › Table S1.docx]

**Table S1.** Accession numbers of grass DEFL sequences in the NCBI (<https://www.ncbi.nlm.nih.gov/>), UniProt (<https://www.uniprot.org/>), Maize Genetics and Genomics (<https://www.maizegdb.org/>) databases**.**

***Aegilops tauschii* ssp*. tauschii*:**

**NCBI**

XP_020164944.1

XP_020181663.1

**UniProt**

M8AXA7

M8AY16

M8BBJ7

M8BCH3

M8BLA1

M8BLJ3

M8BLN5

M8BWE5

M8BX19

M8CHK5

M8CHL1

M8D1D3

N1QRW8

R7W2L2

R7W4C2

R7W5W7

R7W7L2

R7W8W0

R7WCE5

***Aegilops tauschii* ssp. *strangulata*:**

**UniProt**

A0A452XDC0

A0A452XJD0

A0A452XJF3

A0A452XJF4

A0A452XJH5

A0A452XJH7

A0A452XJL3

A0A452XR74

A0A452XR94

A0A452XRA5

A0A453ADC6

A0A453CHF3

A0A453GEA6

A0A453GEC4

A0A453GEM8

A0A453J2T0

A0A453LGD8

A0A453LGP5

A0A453LGR4

A0A453N2B5

A0A453R5R7

A0A453R5K9

A0A453R5Q9

***Avena sativa*:**

**NCBI**

AYU75326.1

**UniProt**

A0A223FQC9

A0A223FQE1

A0A286L6T1

A0A2L0U0E9

A0A2L0U0E7

A0A2L0U0J1

A0A2L0U0N0

A0A2L0U0S0

A0A2L0U0W0

A0A2L0U0X2

A0A2L0U0X5

***Brachypodium distachyon*:**

**NCBI**

KQJ85837.1

XP_003573541

XP_003573542.1

**UniProt**

A0A0Q3GLW6

A0A0Q3E274

A0A0Q3KID4

A0A0Q3LKK6

A0A0Q3LM68

A0A2K2DRX0

A0A2K2DHP2

I1IBT1

I1IRM1

I1IW23

***Hordeum vulgare*:**

**NCBI**

AAB01671.1

BAK04559.1

BAK07467.1

KAE8804473.1

KAE8814413.1

**UniProt**

F2CTD8

F2D2I3

M0UGL3

M0V6B0

M0WF11

M0X4M0

M0XKY4

M0Y045

M0Z6T6

A0A287IVV9

A0A287PZ38

A0A287PZ61

A0A287RV77

***Zea mays*:**

**NCBI**

ACG25630.1

CEJ09690.1

NP_001146963.1

**Maize Genetics and Genomics Database**

AC206262.3_FGP004

AC208126.3_FGP001

AC209356.4_FGP001

AC211996.4_FGP002

GRMZM2G149869_P01

GRMZM2G449327_P01

GRMZM2G136771_P01

GRMZM2G040932_P01

GRMZM2G055621_P01

GRMZM2G079962_P01

GRMZM2G097084_P01

GRMZM2G426158_P01

GRMZM2G050994_P01

GRMZM2G179854_P01

GRMZM2G079547_P01

GRMZM2G101584_P01

GRMZM2G054658_P01

GRMZM2G097719_P01

GRMZM2G004354_P01

GRMZM2G047842_P01

**UniProt**

A0A0A1P1P4

A0A1D6N294

A0A1D6PQA3

A0A317Y7J2

A0A3L6DAF8

A0A3L6DFJ2

A0A3L6DWC4

A0A3L6EKT9

A0A3L6EJV4

A0A3L6ES27

A0A3L6F4B4

A0A3L6F4Y6

A0A3L6F5E3

A0A3L6F5J7

A0A3L6FIN6

A0A3L6FT27

A0A3L6FTF1

A0A3L6G551

A0A3L6G6X8

B3WFQ7

B4FVX5

B6SJ49

B6SJ50

B6SJE6

B6SJI6

B6SMX5

B6SQK6

B6T0W9

B6T664

B6TP10

B6UHE2

C5JA66

D1MAH4

K7W037

***Panicum hallii*:**

**NCBI**

PAN04253.1

PAN04256.1

PUZ74278.1

XP_025795939.1

XP_025796043.1

XP_025800124.1

XP_025819879.1

XP_025822684.1

XP_025824714.1

XP_025825571.1

XP_025826397.1

XP_025826920.1

**UniProt**

A0A2T7CI49

A0A2T7CLJ7

A0A2T7CSH4

A0A2T7CXK4

A0A2T7CXK9

A0A2T7D4K0

A0A2T7D4P7

A0A2T7DA42

A0A2T7DA56

A0A2T7EMF4

A0A2T7F598

A0A2T7F9B1

A0A2S3I4B3

A0A2S3ID96

A0A2S3IP19

***Panicum miliaceum*:**

**NCBI**

RLM79195.1

**UniProt**

A0A3L6PPJ9

A0A3L6PSZ4

A0A3L6RMQ6

A0A3L6QAT2

A0A3L6QDN3

A0A3L6QEN7

A0A3L6QG63

A0A3L6QJ08

A0A3L6QKH5

A0A3L6S3Y1

A0A3L6TFE0

A0A3L6TFI8

A0A3L6TFQ3

A0A3L6TL65

A0A3L6TV62

***Setaria italica*:**

**NCBI**

RCV05284.1

RCV10021.1

RCV13607.1

RCV13608.1

RCV13609.1

RCV18918.1

RCV18919.1

RCV18916.1

RCV38346.1

RCV39228.1

RCV39520.1

RCV39534.1

RCV43523.1

XP_004953176.1

XP_022684422.1

**UniProt**

A0A368QGN4

A0A368QKA8

A0A368SNM1

A0A368SQI9

A2TH14

K4AHF3

K3YB96

K3YBA3

K3YBC9

K3YBZ2

K3YLP8

K3YLU9

K3YX99

K3YXA4

K3ZYN4

***Setaria viridis*:**

**NCBI**

TKW02011.1

TKW02398.1

TKW28860.1

TKW28862.1

TKW28863.1

TKW31067.1

**UniProt**

A0A4U6T131

A0A4U6T440

A0A4U6T918

A0A4U6TIE6

A0A4U6TPX6

A0A4U6TRS5

A0A4U6TS57

A0A4U6U2U7

A0A4U6U435

A0A4U6VF45

A0A4U6W0K0

A0A4U6W1U2

A0A4U6W280

A0A4U6W8N8

A0A4U6WCH8

A0A4U6WGS5

***Sorghum bicolor*:**

**NCBI**

KXG23312.1

KXG34059.1

KXG34060.1

KXG34062.1

KXG34064.1

KXG39004.1

OQU88161.1

XP_002446148.1

XP_002446801.1

XP_002451983.1

XP_002452484.1

XP_002452485.1

XP_002459603.1

XP_002459604.1

XP_002459605.1

XP_002460885.1

XP_002468581.1

XP_021316280.1

XP_021317480.1

XP_021321511.1

**UniProt**

A0A1B6PTG6

A0A1B6PTK8

A0A1W0W7P4

A0A1W0VUC9

A0A1Z5R8J3

A0A1Z5RLB6

A0A1Z5S8G4

A0A1Z5S8Q7

C5XM93

***Triticum aestivum*:**

**NCBI**

AIA66988.1

AIA67001.1

AIA67007.1

AIA67010.1

AIA67016.1

**UniProt**

A0A060AJ86

A0A060AJ92

A0A060AJA7

A0A060AJT9

A0A060AJU9

A0A060APV2

A0A060AQ88

A0A341Y8Y8

A0A3B6APZ4

A0A3B6ARD1

A0A3B6CCE0

A0A3B6D513

A0A3B6D552

A0A3B6D5H0

A0A3B6EP91

A0A3B6EQ78

A0A3B6ET50

A0A3B6FVY6

A0A3B6FYV1

A0A3B6H468

A0A3B6IY35

A0A3B6IYR0

A0A3B6IZN5

A0A3B6JRE0

A0A3B6KTT0

A0A3B6MUH5

A0A3B6MVI8

A0A3B6NKL7

A0A3B6PIU0

A0A3B6RBM2

A0A3B6RFT4

A0A3B6SA89

A0A3B6SN90

A0A3B6TG12

A0A3B6TMC3

A0A3B5XU39

A0A3B5XUE9

A0A3B5YQ69

A0A3B5YQ71

A0A3B5YR60

A0A3B5YRZ2

A0A3B5YT18

A0A3B5ZMC6

A0A3B5ZP16

A0A3B5ZPZ7

A0A3B5ZQC2

A9UID9

A9UIE0

C9E1C1

W4ZVB1

W4ZVX9

W4ZWJ1

W4ZXA3

W5A8J5

W5A907

W5ALK8

W5AMD3

W5BI32

W5BMD8

W5BY35

W5CTA6

W5DB41

W5EHZ3

W5HIB6

***Triticum turgidum*:**

**NCBI**

VAI10189.1

VAI24635.1

VAI35182.1

**UniProt**

A0A446V4D6

A0A446W294

A0A446MIX6

A0A446KH17

A0A446TAV9

A0A446V928

A0A446W739

A0A446VA72

A0A446W8E4

A0A446KIL8

A0A446LTF6

A0A446KIQ0

A0A446LTE6

A0A446SGG4

A0A446SGF8

A0A446P192

A0A446P1L4

A0A446P1R7

A0A446QBK2

A0A446IHS9

A0A446JGG7

A0A446JGJ0

A0A446YKS4

A0A446IK51

A0A446IK37

A0A446JJ68

A0A446IK54

A0A446JJA0

C9E1C2

C9E1C3

C9E1C4

C9E1C5

C9E1C6

C9E1C7

***Triticum urartu*:**

**NCBI**

EMS52097.1

EMS52103.1

EMS52277.1

EMS54457.1

EMS54459.1

EMS59557.1

EMS65298.1

**UniProt**

M7YF55

M7YZB1

M7YZR7

M7ZMM4

M7ZZ14

M8A0P3

M8AUC6

T1LVE3

T1NV13
