# Supplementary material for: Defensins of Grasses: A Systematic Review
Source: Biomolecules. 2020 Jul 10;10(7):1029. doi: 10.3390/biom10071029 (PMC7407236; doi:10.3390/biom10071029)
Supplement: Supplementary file 1 [file biomolecules-10-01029-s001.zip › Table S2.docx]

**Table S2.** Genome characteristics and DEFL number identified by *in silico* mining in grass species.

| Species | Chromosome no. | Genome size (1C) | DEFLs no. | Reference |
| --- | --- | --- | --- | --- |
| Pooideae, Triticeae | | | | |
| *Aegilops tauschii* ssp*. tauschii* Coss. | 2x=14 | 4,33 Gb [128] | 21 | our data |
| *Aegilops tauschii* ssp. *strangulata* Coss. | 2x=14 | 4,3 Gb [129] | 23 | our data |
| *Triticum aestivum* L. | 6x=42 | 15,4 Gb [130] | 69^#^ | our data |
| *Triticum kiharae* Dorof. et Migush. | 6x=42 | ‒ | 60* | [84] |
| *Triticum turgidum* L. | 4x=28 | 10,1 Gb [131] | 37 | our data |
| *Triticum urartu* Thum. ex Gandilyan | 2x=14 | 4,9 Gb [132] | 16 | our data |
| *Leymus arenarius* (L.) Hochst. | 8x=56 | ‒ | 7* | [27] |
| *Hordeum vulgare* L. | 2x=14 | 4,79 Gb [133] | 18^#^ | our data |
| Pooideae, Aveneae | | | | |
| *Avena sativa* L. | 6x=42 | 12,3 Gb [134] | 12^#^ | our data |
| Pooideae, Brachypodeae | | | | |
| *Brachypodium distachyon* (L.) P.Beauv. | 2x=10 | 271,3 Mb [135] | 13 | our data |
| Oryzoideae, Oryzeae | | | | |
| *Oryza sativa* L. | 2x=24 | 388,8 Mb [136] | 41 | [38] |
| Panicoideae, Paniceae | | | | |
| *Panicum hallii* Vasey | 2x=18 | 484,6 Mb [110] | 27 | our data |
| *Panicum miliaceum* L. | 4x=36 | 854,8 Mb [137] | 16 | our data |
| *Setaria italica* (L.) P.Beauv. | 2x=18 | 400,9 Mb [138] | 30^#^ | our data |
| *Setaria viridis* (L.) P.Beauv. | 2x=18 | 397,0 Mb ^1^ | 22 | our data |
| Panicoideae, Andropogoneae | | | | |
| *Sorghum bicolor* (L.) Moench | 2x=20 | 730 Mb [39] | 29 | our data |
| *Zea mays* L. | 2x=20 | 2,1 Gb [140] | 57^#^ | our data |

^1^ Representative genome of *Setaria viridis* (assembly ASM1293433v1) submitted to the GenBank database 2020/04/30.

^#^ ‒ genome and transcriptome data, * ‒ transcriptome data, without any sign ‒ genome data.

1. Zimin, A.V.; Puiu, D.; Luo, M.C.; Zhu, T.; Koren, S.; Marçais, G.; Yorke, J.A.; Dvořák, J.; Salzberg, S.L. Hybrid assembly of the large and highly repetitive genome of *Aegilops tauschii*, a progenitor of bread wheat, with the MaSuRCA mega-reads algorithm. *Genome Res.* **2017**, *27*, 787‒792, doi:10.1101/gr.213405.116.
2. Luo, M.C.; Gu, Y.Q.; Puiu, D.; Wang, H.; Twardziok, S.O.; Deal, K.R.; Huo, N.; Zhu, T.; Wang, L.; Wang, Y.; et al. Genome sequence of the progenitor of the wheat D genome Aegilops tauschii. *Nature* **2017**, *551*, 498‒502, doi:10.1038/nature24486.
3. Zimin, A.V.; Puiu, D.; Hall, R.; Kingan, S.; Clavijo, B.J.; Salzberg, S.L. The first near-complete assembly of the hexaploid bread wheat genome, *Triticum aestivum*. *Gigascience* **2017**, *6*, 1‒7, doi:10.1093/gigascience/gix097.
4. Avni, R.; Nave, M.; Barad, O.; Baruch, K.; Twardziok, S.O.; Gundlach, H.; Hale, I.; Mascher, M.; Spannagl, M.; Wiebe, K.; et al. Wild emmer genome architecture and diversity elucidate wheat evolution and domestication. *Science* **2017**, *357*, 93–97, doi:10.1126/science.aan0032.
5. Ling, H.Q.; Ma, B.; Shi, X.; Liu, H.; Dong, L.; Sun, H.; Cao, Y.; Gao, Q.; Zheng, S.; Li, Y.; et al. Genome sequence of the progenitor of wheat A subgenome *Triticum urartu*. *Nature* **2018**, *557*, 424–428, doi:10.1038/s41586-018-0108-0.
6. Mascher, M.; Gundlach, H.; Himmelbach, A.; Beier, S.; Twardziok, S.O.; Wicker, T.; Radchuk, V.; Dockter, C.; Hedley, P.E.; Russell, J.; et al. A chromosome conformation capture ordered sequence of the barley genome. *Nature* **2017**, *544*, 427–433, doi:10.1038/nature22043.
7. Yan, H.; Martin, S.L.; Bekele, W.A.; Latta, R.G.; Diederichsen, A.; Peng, Y.; Tinker, N.A. Genome size variation in the genus *Avena*. *Genome* **2016**, *59*, 209‒220, doi:10.1139/gen-2015-0132.
8. International Brachypodium Initiative. Genome sequencing and analysis of the model grass *Brachypodium distachyon*. *Nature* **2010**, *463*, 763‒768, doi:10.1038/nature08747.
9. International Rice Genome Sequencing Project. The Map-Based Sequence of the Rice Genome. *Nature* **2005**, *436*, 793–800, doi:10.1038/nature03895.
10. Zou, C.; Li, L.; Miki, D.; Li, D.; Tang, Q.; Xiao, L.; Rajput, S.; Deng, P.; Peng, L.; Jia, W.; et al. The genome of broomcorn millet. *Nat. Commun.* **2019**, *10*, 436, doi:10.1038/s41467-019-08409-5.
11. Bennetzen, J.L.; Schmutz, J.; Wang, H.; Percifield, R.; Hawkins, J.; Pontaroli, A.C.; Estep, M.; Feng, L.; Vaughn, J.N.; Grimwood, J.; et al. Reference genome sequence of the model plant Setaria. *Nat. Biotechnol.* **2012**, *30*, 555–561, doi:10.1038/nbt.2196.
12. Paterson, A.; Bowers, J.; Bruggmann, R.; Dubchak, I.; Grimwood, J.; Gundlach, H.; Haberer, G.; Hellsten, U.; Mitros, T.; Poliakov, A.; et al. The *Sorghum bicolor* genome and the diversification of grasses. *Nature* **2009**, *457*, 551–556, doi:10.1038/nature07723.
13. Jiao, Y.; Peluso, P.; Shi, J.; Liang, T.; Stitzer, M.C.; Wang, B.; Campbell, M.S.; Stein, J.C.; Wei, X.; Chin, C.S.; et al. Improved maize reference genome with single-molecule technologies. *Nature* **2017**, *546*, 524–527, doi:10.1038/nature22971.
