# Supplementary material for: Defensins of Grasses: A Systematic Review
Source: Biomolecules. 2020 Jul 10;10(7):1029. doi: 10.3390/biom10071029 (PMC7407236; doi:10.3390/biom10071029)
Supplement: Supplementary file 1 [file biomolecules-10-01029-s001.zip › Table S3.docx]

**Table S3.** DEFLs of one species with identical mature peptides.

| **№** | **Name of sequences with identical mature peptides** | **Name in the tree (Figure 3)** |
| --- | --- | --- |
| ***T. kiharae* (TK)** | | |
| 1 | DEFL1-3, DEFL1-4 | DEFL1-3_TK* |
| 2 | DEFL1-6, DEFL1-7, DEFL1-8, DEFL1-9 | DEFL1-6_TK* |
| 3 | DEDFL1-17, DEFL1-18, DEFL1-19, DEFL1-20, DEFL1-21 | DEFL1-17_TK* |
| 4 | DEFL1-25, DEFL1-26, DEFL1-27, DEFL1-28 | DEFL1-25_TK* |
| 5 | DEFL1-31, DEFL1-40 | DEFL1-31_TK* |
| 6 | DEFL1-36, DEFL1-46, DEFL1-50 | DEFL1-36_TK* |
| 7 | DEFL1-37, DEFL1-38, DEFL1-39 | DEFL1-37_TK* |
| 8 | DEFL1-51, DEFL1-52 | DEFL1-51_TK* |
| ***T. turgium* (TRITD)** | | |
| 9 | A0A446V4D6, A0A446W294 | A0A446V4D6_TRITD* |
| ***T. aestivum* (WHEAT)** | | |
| 10 | γ_1_-purothionin, AIA67016.1 | G1-purothionin_WHEAT* |
| 11 | γ_2_-purothionin, AIA67007.1 | G2-purothionin_WHEAT* |
| 12 | A0A3B6IYR0, AIA67010.1 | A0A3B6IYR0_WHEAT* |
| 13 | A0A341Y8Y8, A0A3B6RFT4 | A0A341Y8Y8_WHEAT* |
| 14 | W5A8J5, A0A060AJ92, A0A3B5ZQC2 | W5A8J5_WHEAT* |
| 15 | A0A3B6NKL7, A9UID9, A9UIE0 | A0A3B6NKL7_WHEAT* |
| 16 | W4ZXA3, W5AMD3 | W4ZXA3_WHEAT* |
| 17 | A0A3B5YQ71, A0A3B5YR60 | A0A3B5YQ71_WHEAT* |
| ***A. tauschii* ssp. *strangulata* (AEGTS)** | | |
| 18 | A0A452XJD0, A0A452XJH7 | A0A452XJD0_AEGTS* |
| 19 | A0A452XR94, A0A452XRA5 | A0A452XR94_AEGTS* |
| ***A. sativa* (AVESA)** | | |
| 20 | AYU75326.1, A0A2L0U0X2 | AYU75326.1_AVESA* |
| ***H. vulgare* (HORVV)** | | |
| 21 | BAK07467.1, M0X4M0 | BAK07467.1_HORVV* |
| 22 | F2CTD8, M0WF11 | F2CTD8_HORVV* |
| 23 | KAE8814413.1, A0A287IVV9 | KAE8814413.1_HORVV* |
| 24 | W-hordothionin, KAE8804473.1 | W-hordothionin_HORVV* |
| ***S. bicolor* (SORBI)** | | |
| 25 | Sialpha2.1, XP_021321511.1 | Sialpha2.1_SORBI* |
| 26 | Sialpha1, A0A1Z5R8J3 | Sialpha1_SORBI* |
| 27 | XP_021317480.1, XP_002468581.1 | XP_021317480.1_ SORBI* |
| ***P. hallii* (POAL)** | | |
| 28 | PAN04253.1, PUZ74278.1 | PAN04253.1_POAL* |
| 29 | XP_025819879.1, A0A2T7D4K0 | XP_025819879.1_POAL* |
| 30 | A0A2S3I4B3, A0A2T7DA56 | A0A2S3I4B3_POAL* |
| 31 | A0A2T7F598, XP_025796043.1 | A0A2T7F598_POAL* |
| 32 | XP_025824714.1, A0A2T7CXK9 | XP_025824714.1_POAL* |
| 33 | XP_025825571.1, A0A2T7CXK4 | XP_025825571.1_POAL* |
| 34 | XP_025800124.1, A0A2T7EMF4 | XP_025800124.1_POAL* |
| ***Z. mays* (MAIZE)** | | |
| 35 | ZmDEF1, CEJ09690.1, B6SJI6, A0A3L6EKT9 | ZMDEF1_MAIZE* |
| 36 | ZmD32, B6SJ49, B6SJE6 | ZMD32_MAIZE* |
| 37 | ZmES2, ZmES3 | ZMES2_MAIZE* |
| 38 | ZmESR-6, D1MAH4 | ZMESR-6_MAIZE* |
| 39 | γ_2_-zeathionin, NP_001146963.1 | G2-zeathionin_MAIZE* |
| 40 | AC208126.3_FGP001, A0A3L6F5J7, B6SJ50 | AC208126.3_FGP001_MAIZE* |
| 41 | 0A3L6ES27, B6TP10 | 0A3L6ES27_MAIZE* |
| 42 | A0A3L6G6X8, A0A1D6PQA3 | A0A3L6G6X8_MAIZE* |
| 43 | A0A1D6N294, A0A3L6FIN6 | A0A1D6N294_MAIZE* |
| 44 | A0A3L6EJV4, B6T664 | A0A3L6EJV4_MAIZE* |
| 45 | A0A3L6F4B4, B6SQK6 | A0A3L6F4B4_MAIZE* |
| 46 | A0A3L6F4Y6, A0A3L6F5E3, B4FVX5, B6UHE2 | A0A3L6F4Y6_MAIZE* |
| ***L. arenarius* (LA)** | | |
| 47 | DEFL5-4, DEFL5-5 | DEFL5-4_LA* |
