# Supplementary material for: Defensins of Grasses: A Systematic Review
Source: Biomolecules. 2020 Jul 10;10(7):1029. doi: 10.3390/biom10071029 (PMC7407236; doi:10.3390/biom10071029)
Supplement: Supplementary file 1 [file biomolecules-10-01029-s001.zip › Table S4.docx]

**Table S4.** DEFLs of different species with identical mature peptides.

| **№** | **Name of sequences with identical mature peptides** | **Name in the tree (Figure 3)** |
| --- | --- | --- |
| 1 | DEFL1-14_TK, EMS59557.1_TRIUA | DEFL1-14_TK_X |
| 2 | PAN04256.1_POAL, RLM79195.1_PANMI | PAN04256.1_POAL_X |
| 3 | A0A446VA72_TRITD, M7YZB1_TRIUA | A0A446VA72_TRITD_X |
| 4 | RCV13607.1_SETIT, A0A4U6W0K0_SETVI | RCV13607.1_SETIT_X |
| 5 | RCV13609.1_SETIT, A0A4U6W280_SETVI | RCV13609.1_SETIT_X |
| 6 | ZMDEF1_MAIZE*, A2TH14_SETIT | ZMDEF1_MAIZE*_X |
| 7 | A0A4U6WCH8_SETVI, K3YXA4_SETIT | A0A4U6WCH8_SETVI_X |
| 8 | A0A2T7F598_POAL*, A0A3L6QEN7_PANMI | A0A2T7F598_POAL*_X |
| 9 | A0A4U6W8N8_SETVI, K3YX99_SETIT | A0A4U6W8N8_SETVI_X |
| 10 | TAD1_WHEAT, EMS52103.1_D2_TRIUA, A0A446IHS9_TRITD | TAD1_WHEAT_X |
| 11 | DEFL1-23_TK, A0A452XJD0_AEGTS*, F2D2I3_HORVV, M8CHK5_AEGTA | DEFL1-23_TK_X |
| 12 | DEFL1-31_TK, A0A446JGG7_TRITD, W5A907_WHEAT | DEFL1-31_TK_X |
| 13 | R7W5W7_AEGTA, A0A452XJF3_AEGTS, W4ZXA3_WHEAT* | R7W5W7_AEGTA_X |
| 14 | M8AY16_AEGTA, A0A452XR94_AEGTS*, W5A8J5_WHEAT* | M8AY16_AEGTA_X |
| 15 | DEFL1-51_TK*, A0A060AJ86_WHEAT | DEFL1-51_TK*_X |
| 16 | W4ZVX9_WHEAT, A0A446IK37_TRITD | W4ZVX9_WHEAT_X |
| 17 | C9E1C1_WHEAT, A0A452XR74_AEGTS, M8AXA7_AEGTA | C9E1C1_WHEAT_X |
| 18 | C9E1C2_TRITD, M7ZMM4_TRIUA, W4ZVB1_WHEAT | C9E1C2_TRITD_X |
| 19 | R7W2L2_AEGTA, A0A3B5ZPZ7_WHEAT | R7W2L2_AEGTA # |
| 20 | DEFL1-1_TK, A0A3B6NKL7_WHEAT*, A0A446V4D6_TRITD*, A0A453N2B5_AEGTS | DEFL1-1_TK_X |
| 21 | C9E1C4_TRITD, A0A3B6PIU0_WHEAT | C9E1C4_TRITD_X |
| 22 | DEFL1-2_TK, A0A3B5ZMC6_WHEAT, A0A452XJL3_AEGTS, M8BLN5_AEGTA | DEFL1-2_TK_X |
| 23 | Tk-AMP-D1_TK, EMS52097.1_ TRIUA, A0A3B5XUE9_WHEAT | Tk-AMP-D1_TK_X |
| 24 | A0A3B6SN90_WHEAT, A0A446YKS4_TRITD | A0A3B6SN90_WHEAT_X |
| 25 | A0A3B5YQ69_WHEAT, A0A446JGJ0_TRITD | A0A3B5YQ69_WHEAT_X |
| 26 | A0A452XJH5_AEGTS, M8BLA1_AEGTA, W5ALK8_WHEAT | A0A452XJH5_AEGTS_X |
| 27 | A0A3B5ZP16_WHEAT, A0A452XJF4_AEGTS, M8CHL1_AEGTA | A0A3B5ZP16_WHEAT_X |
| 28 | K3YB96_SETIT, A0A4U6TS57_SETVI | K3YB96_SETIT_X |
| 29 | K3YBZ2_SETIT, A0A4U6TRS5_SETVI | K3YBZ2_SETIT_X |
| 30 | DEFL1-44_TK, A0A3B6CCE0_WHEAT, A0A446MIX6_TRITD | DEFL1-44_TK_X |
| 31 | DEFL1-33_TK, W5BY35_WHEAT | DEFL1-33_TK_X |
| 32 | K4AHF3_SETIT, A0A4U6T918_SETVI | K4AHF3_SETIT_X |
| 33 | DEFL1-15_TK, A0A060AJU9_WHEAT, A0A453J2T0_AEGTS | DEFL1-15_TK_X |
| 34 | DEFL1-16_TK, DEFL3-2_LA, EMS65298.1_TRIUA, A0A3B6IY35_WHEAT, A0A446SGG4_TRITD | DEFL1-16_TK_X |
| 35 | VAI24635.1_TRITD, A0A3B6IYR0_WHEAT*, A0A453J2F3_AEGTS, XP_020164944.1_AEGTA | VAI24635.1_TRITD_X |
| 36 | M7ZZ14_TRIUA, A0A060APV2_WHEAT | M7ZZ14_TRIUA_X |
| 37 | N1QRW8_AEGTA, W5EHZ3_WHEAT | N1QRW8_AEGTA_X |
| 38 | K3ZYN4_SETIT, A0A4U6W1U2_SETVI | K3ZYN4_SETIT_X |
| 39 | Tk-AMP-D1.1_TK, EMS52277.1_ TRIUA, A0A3B6EP91_WHEAT | Tk-AMP-D1.1_TK_X |
| 40 | Tk-AMP-D3_TK, A0A3B6H468_WHEAT, A0A453GEM8_AEGTS, M8BLJ3_AEGTA | Tk-AMP-D3_TK_X |
| 41 | A0A060AQ88_WHEAT, A0A446P192_TRITD | A0A060AQ88_WHEAT_X |
| 42 | Tk-AMP-D4_TK, EMS54457.1_ TRIUA, W5HIB6_WHEAT | Tk-AMP-D4_TK_X |
| 43 | M8BCH3_AEGTA, A0A3B6TMC3_WHEAT, A0A453R5Q9_AEGTS | M8BCH3_AEGTA_X |
| 44 | Tk-AMP-D5_TK, EMS54459.1_ TRIUA, A0A3B6RBM2_WHEAT | Tk-AMP-D5_TK_X |
| 45 | Tk-AMP-D6_TK, A0A3B6TG12_WHEAT, A0A453R5R7_AEGTS, M8BBJ7_AEGTA | Tk-AMP-D6_TK_X |
| 46 | Tk-AMP-D6.1_TK, A0A3B6SA89_WHEAT | Tk-AMP-D6.1_TK_X |
| 47 | M8BX19_AEGTA, A0A341Y8Y8_WHEAT*, A0A453R5K9_AEGTS | M8BX19_AEGTA_X |
| 48 | A0A3B6ET50_WHEAT, A0A446P1R7_TRITD | A0A3B6ET50_WHEAT_X |
| 49 | A0A3B6FYV1_WHEAT, A0A446QBK2_TRITD | A0A3B6FYV1_WHEAT_X |
| 50 | W5DB41_WHEAT, A0A453GEA6_AEGTS | W5DB41_WHEAT_X |
| 51 | A0A3B6EQ78_WHEAT, A0A446P1L4_TRITD | A0A3B6EQ78_WHEAT_X |
| 52 | M8BWE5_AEGTA, A0A453GEC4_AEGTS | M8BWE5_AEGTA_X |
| 53 | A0A4U6WGS5_SETVI, XP_004953176.1_SETIT | A0A4U6WGS5_SETVI_X |
| 54 | A0A368SQI9_SETIT, A0A4U6T131_SETVI | A0A368SQI9_SETIT_X |
| 55 | A0A368QKA8_SETIT, A0A4U6VF45_SETVI | A0A368QKA8_SETIT_X |
| 56 | A0A368SNM1_SETIT, A0A4U6T440_SETVI | A0A368SNM1_SETIT_X |
| 57 | RCV10021.1_SETIT, TKW31067.1_SETVI | RCV10021.1_SETIT_X |
| 58 | RCV18918.1_SETIT, TKW28862.1_SETVI | RCV18918.1_SETIT_X |
| 59 | R7W7L2_AEGTA, A0A453LGD8_AEGTS, A0A453LGP5_AEGTS | R7W7L2_AEGTA_X |
| 60 | A0A3B6ARD1_WHEAT, A0A446KIL8_TRITD | A0A3B6ARD1_WHEAT_X |
| 61 | M8D1D3_AEGTA, A0A3B6D552_WHEAT, A0A453ADC6_AEGTS | M8D1D3_AEGTA_X |
| 62 | W5BMD8_WHEAT, A0A446LTF6_TRITD | W5BMD8_WHEAT_X |
| 63 | W5BI32_WHEAT, A0A446LTE6_TRITD | W5BI32_WHEAT_X |
| 64 | A0A3B6D513_WHEAT, A0A452XDC0_AEGTS | A0A3B6D513_WHEAT_X |
| 65 | A0A3B6D5H0_WHEAT, XP_020181663.1_AEGTA | A0A3B6D5H0_WHEAT_X |
| 66 | K3YBC9_SETIT, A0A4U6TPX6_SETVI | K3YBC9_SETIT_X |
| 67 | R7WCE5_AEGTA, A0A3B6JRE0_WHEAT | R7WCE5_AEGTA_X |
| 68 | T1LVE3_TRIUA, A0A3B6KTT0_WHEAT | T1LVE3_TRIUA_X |
| 69 | AIA66988.1_WHEAT, A0A446TAV9_TRITD | AIA66988.1_WHEAT_X |
| 70 | AIA67001.1_WHEAT, VAI35182.1_TRITD | AIA67001.1_WHEAT_X |
| 71 | R7W8W0_AEGTA, A0A3B6MUH5_WHEAT, A0A453LGR4_AEGTS | R7W8W0_AEGTA_X |
| 72 | Sd6_SO, G2-zeathionin_MAIZE* | Sd6_SO_X |
| 73 | A0A3L6PPJ9_PANMI, XP_025819879.1_POAL* | A0A3L6PPJ9_PANMI_X |
| 74 | M7YF55_TRIUA, A0A446V928_TRITD | M7YF55_TRIUA_X |

**Note.** The following abbreviations are used: TK ‒ *Triticum kiharae*, TRITD ‒ *T. turgidum*, TRIUA ‒ *T. urartu*, WHEAT ‒ *T. aestivum*, AEGTS ‒ *Aegilops tauschii* ssp. *strangulata*, AEGTA ‒ *A. tauschii* ssp. *tauschii*, AVESA ‒ *Avena sativa*, SETIT ‒ *Setaria italica*, SETVI ‒ *S. viridis*, POAL ‒ *Panicum hallii*, PANMI ‒ *P. miliaceum*, MAIZE ‒ *Z. mays*, LA ‒ *L. arenarius*, OS ‒ *O. sativa*, HORVV ‒ *H. vulgare*, SORBI ‒ *Sorghum biolor*, BRADI ‒ *B. distachyon*, SO ‒ *Saccharum* spp**.** Sequences marked with * are listed in Table S3.
