# Supplementary figures and images for: Defensins of Grasses: A Systematic Review
Source: Biomolecules. 2020 Jul 10;10(7):1029. doi: 10.3390/biom10071029 (PMC7407236; doi:10.3390/biom10071029)

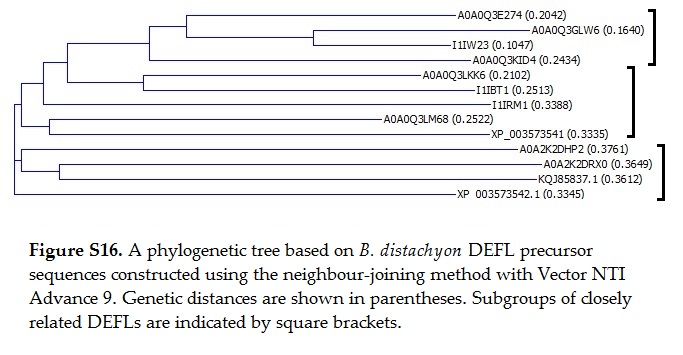

Supplement: Supplementary file 1 [file biomolecules-10-01029-s001.zip › Figure S16.jpg]

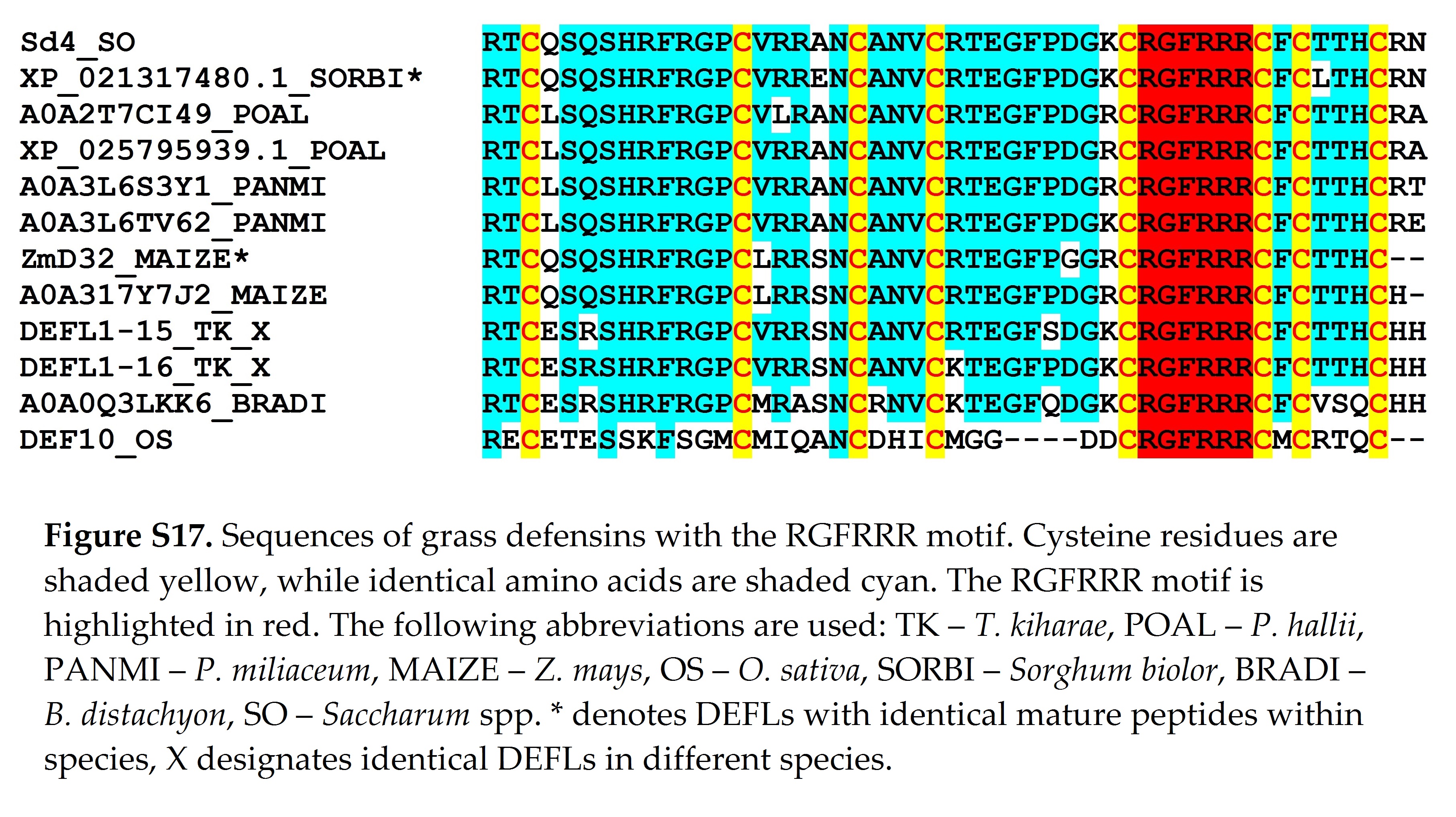

Supplement: Supplementary file 1 [file biomolecules-10-01029-s001.zip › Figure S17.jpg]

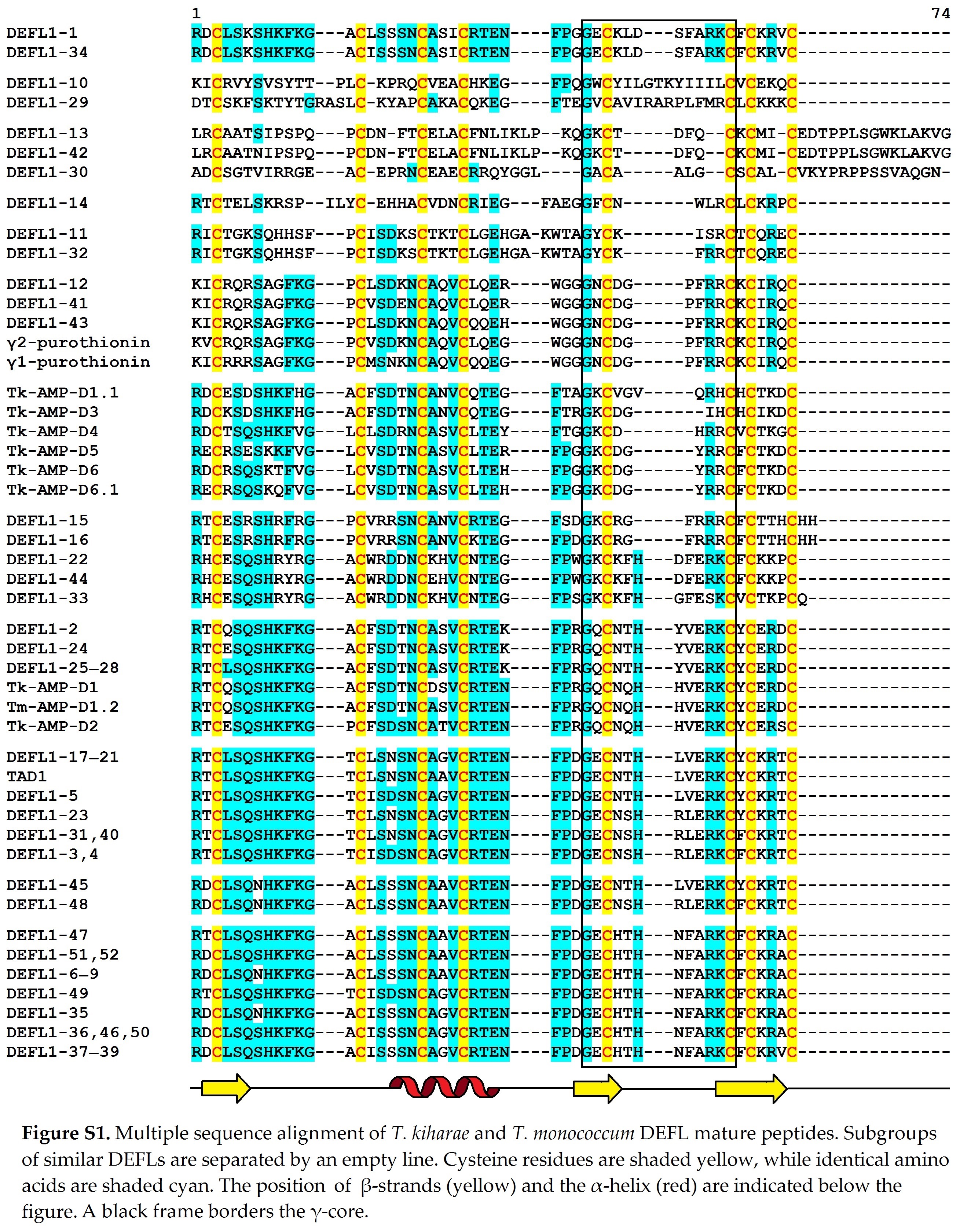

Supplement: Supplementary file 1 [file biomolecules-10-01029-s001.zip › Figure S1.jpg]

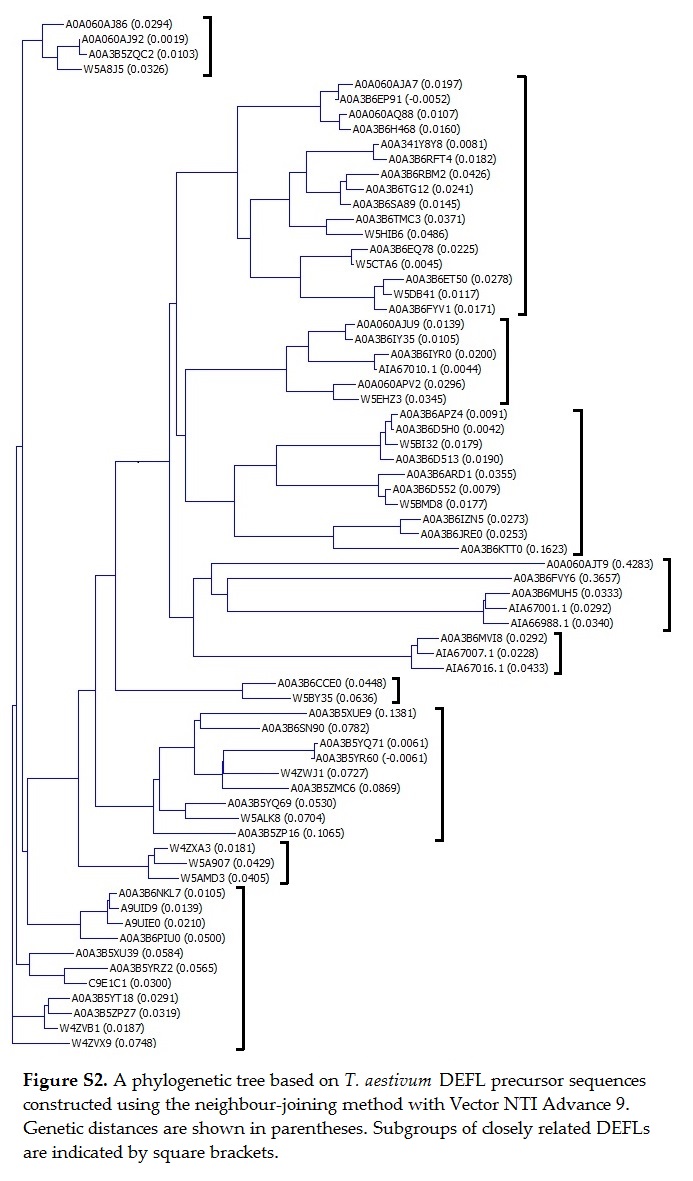

Supplement: Supplementary file 1 [file biomolecules-10-01029-s001.zip › Figure S2.jpg]

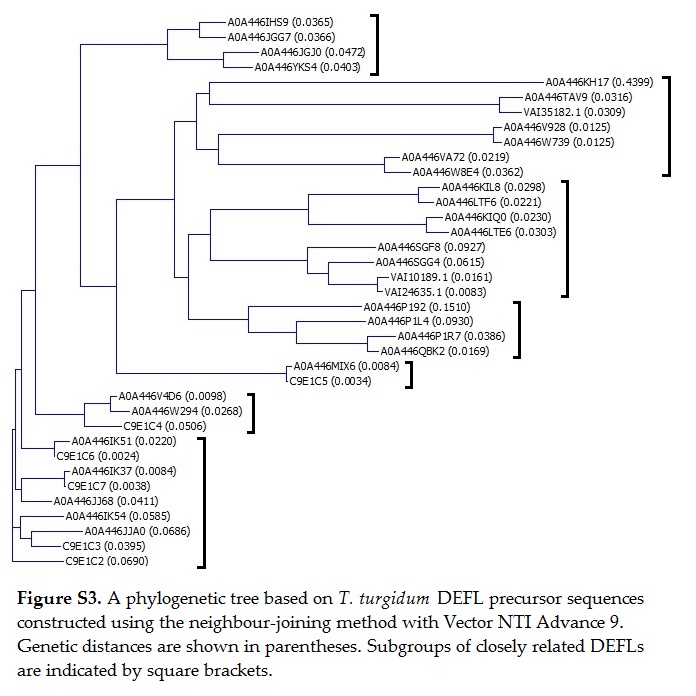

Supplement: Supplementary file 1 [file biomolecules-10-01029-s001.zip › Figure S3.jpg]

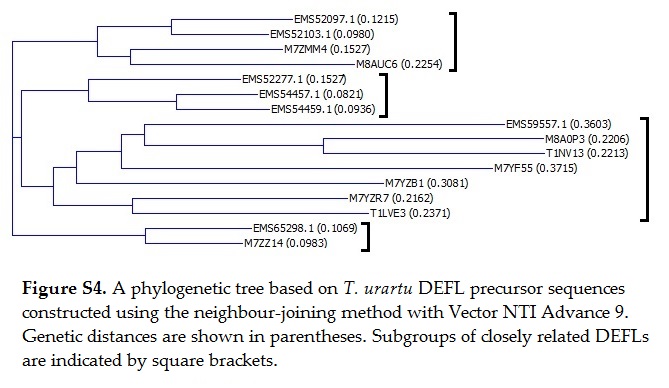

Supplement: Supplementary file 1 [file biomolecules-10-01029-s001.zip › Figure S4.jpg]

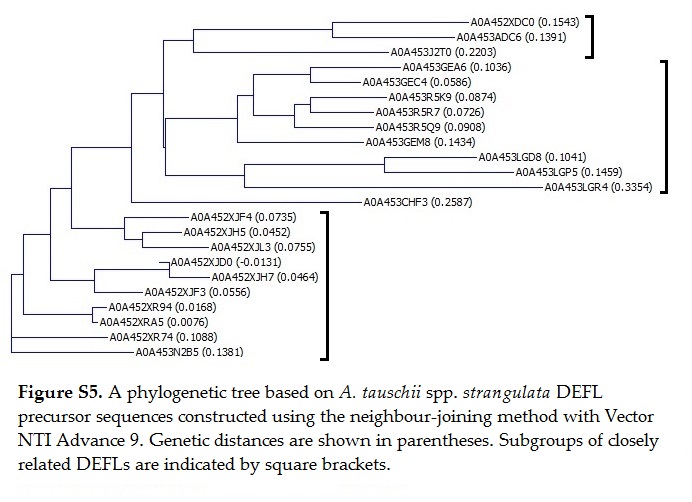

Supplement: Supplementary file 1 [file biomolecules-10-01029-s001.zip › Figure S5.jpg]

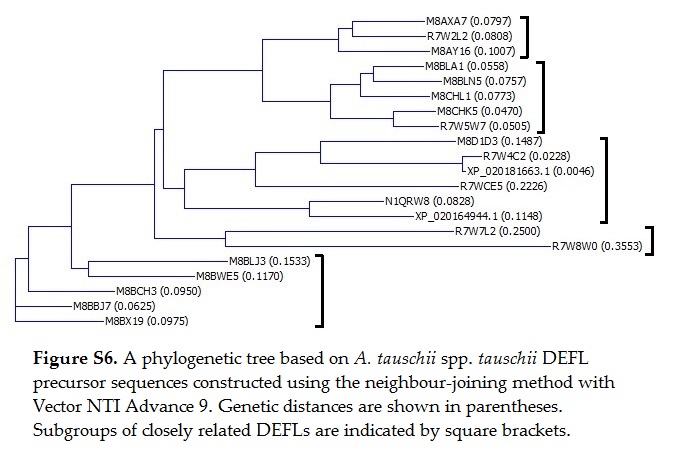

Supplement: Supplementary file 1 [file biomolecules-10-01029-s001.zip › Figure S6.jpg]

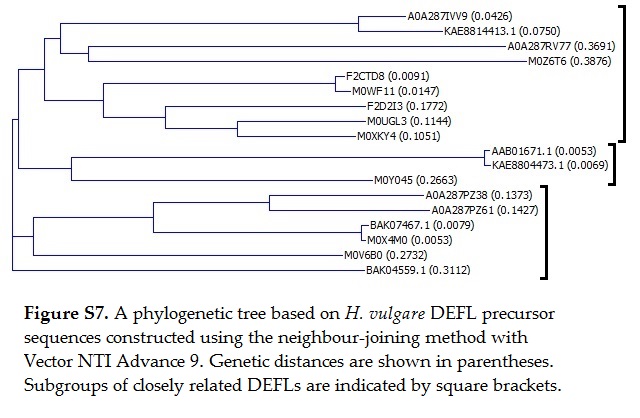

Supplement: Supplementary file 1 [file biomolecules-10-01029-s001.zip › Figure S7.jpg]

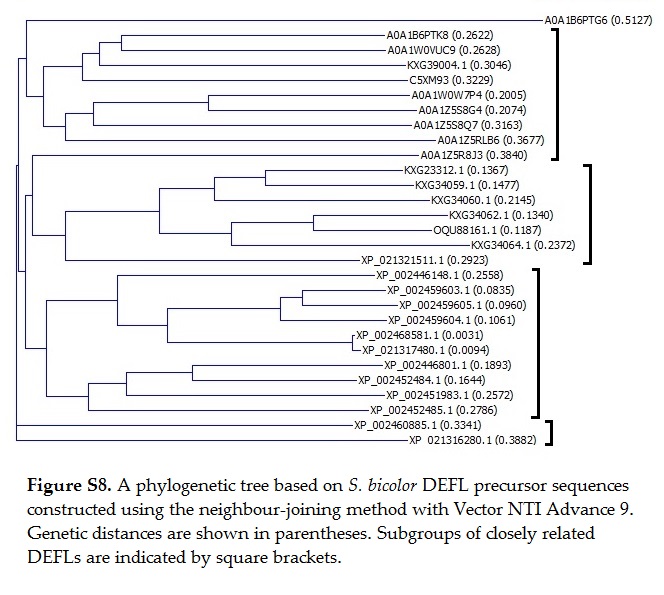

Supplement: Supplementary file 1 [file biomolecules-10-01029-s001.zip › Figure S8.jpg]

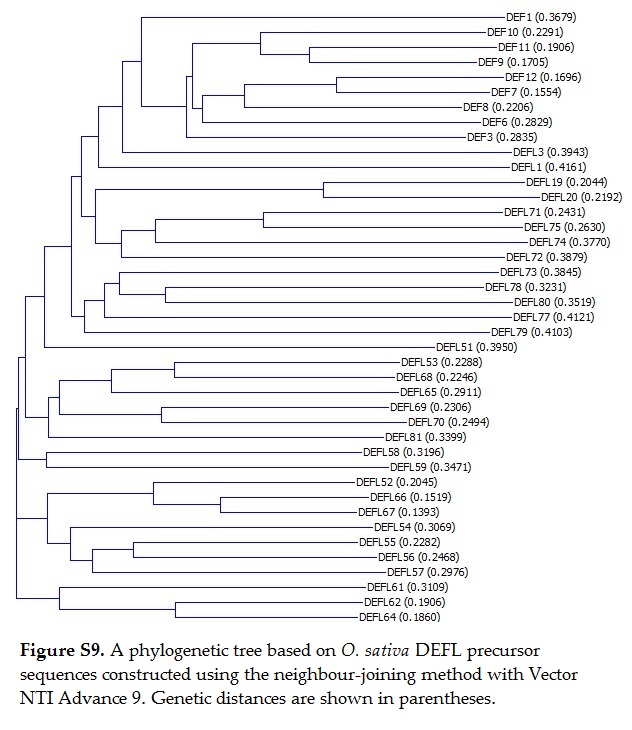

Supplement: Supplementary file 1 [file biomolecules-10-01029-s001.zip › Figure S9.jpg]

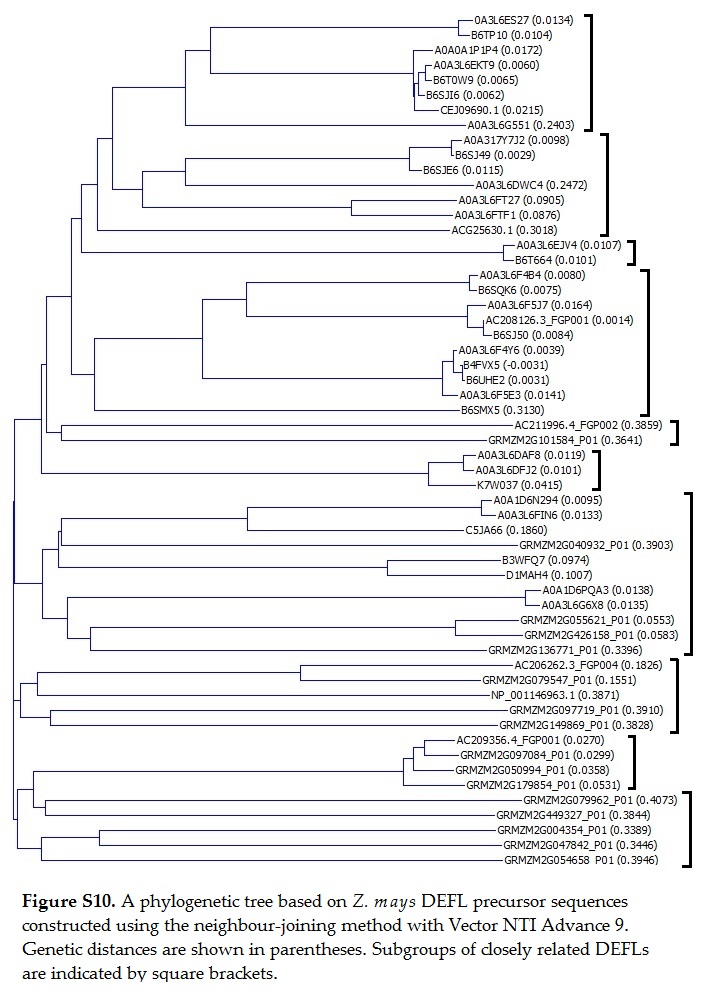

Supplement: Supplementary file 1 [file biomolecules-10-01029-s001.zip › Figure S10.jpg]

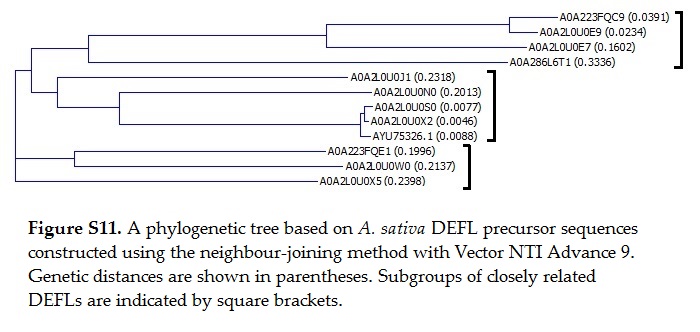

Supplement: Supplementary file 1 [file biomolecules-10-01029-s001.zip › Figure S11.jpg]

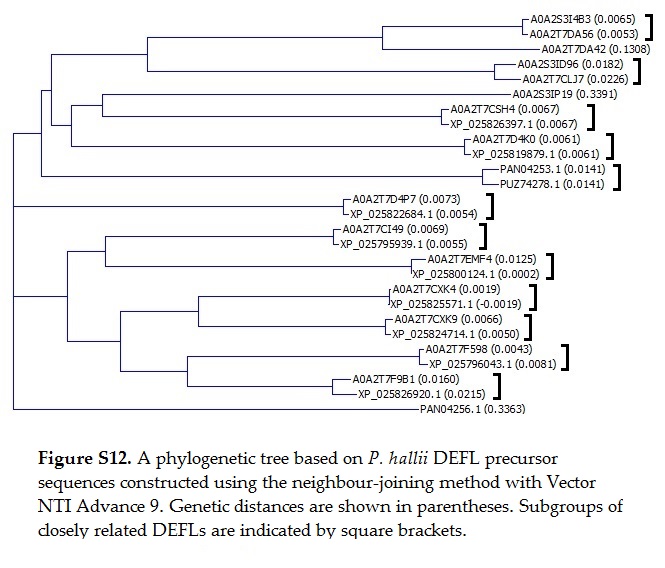

Supplement: Supplementary file 1 [file biomolecules-10-01029-s001.zip › Figure S12.jpg]

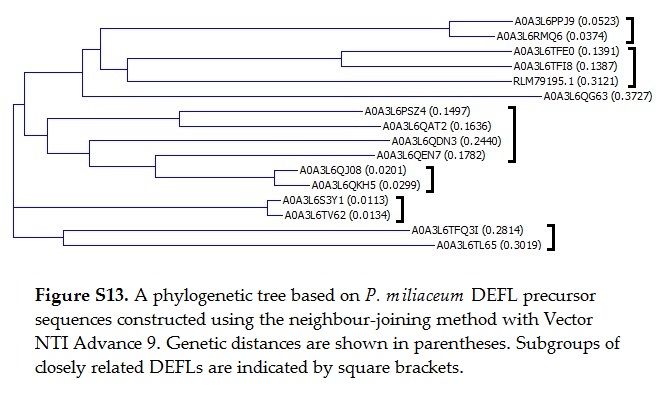

Supplement: Supplementary file 1 [file biomolecules-10-01029-s001.zip › Figure S13.jpg]

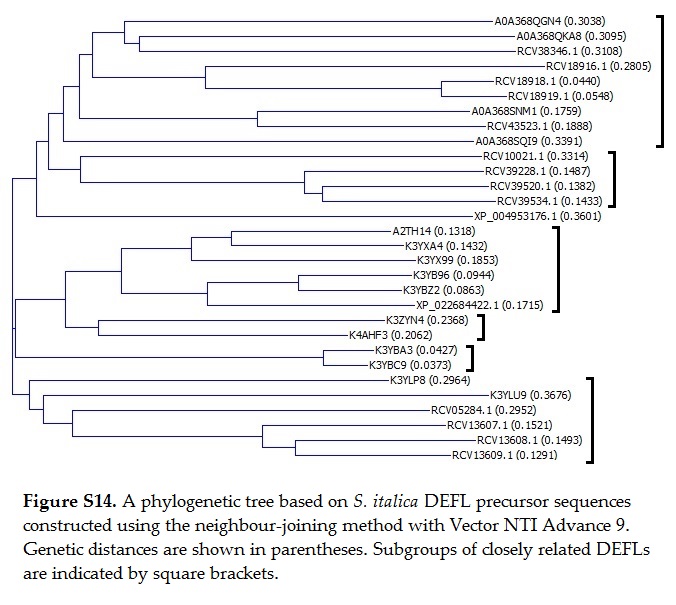

Supplement: Supplementary file 1 [file biomolecules-10-01029-s001.zip › Figure S14.jpg]

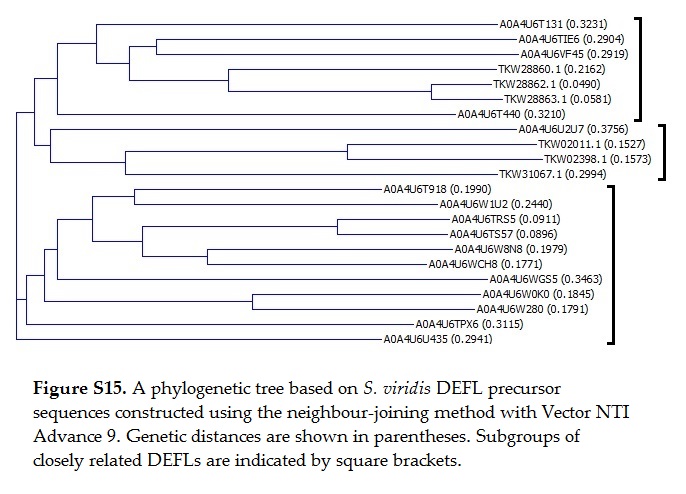

Supplement: Supplementary file 1 [file biomolecules-10-01029-s001.zip › Figure S15.jpg]
